# Potential-induced nanoclustering of metallic catalysts during electrochemical CO_2_ reduction

**DOI:** 10.1038/s41467-018-05544-3

**Published:** 2018-08-06

**Authors:** Jianfeng Huang, Nicolas Hörmann, Emad Oveisi, Anna Loiudice, Gian Luca De Gregorio, Oliviero Andreussi, Nicola Marzari, Raffaella Buonsanti

**Affiliations:** 1 Laboratory of Nanochemistry for Energy (LNCE), Department of Chemical Sciences and Engineering, École Polytechnique Fédérale de Lausanne, CH-1950 Sion, Switzerland; 2 Theory and Simulation of Materials (THEOS), and National Centre for Computational Design and Discovery of Novel Materials (MARVEL), École Polytechnique Fédérale de Lausanne, CH-1015 Lausanne, Switzerland; 3 Interdisciplinary Centre for Electron Microscopy (CIME), École Polytechnique Fédérale de Lausanne, CH-1015 Lausanne, Switzerland; 4 Department of Physics, University of North Texas, Denton, TX-76203 USA

## Abstract

In catalysis science stability is as crucial as activity and selectivity. Understanding the degradation pathways occurring during operation and developing mitigation strategies will eventually improve catalyst design, thus facilitating the translation of basic science to technological applications. Herein, we reveal the unique and general degradation mechanism of metallic nanocatalysts during electrochemical CO_2_ reduction, exemplified by different sized copper nanocubes. We follow their morphological evolution during operation and correlate it with the electrocatalytic performance. In contrast with the most common coalescence and dissolution/precipitation mechanisms, we find a potential-driven nanoclustering to be the predominant degradation pathway. Grand-potential density functional theory calculations confirm the role of the negative potential applied to reduce CO_2_ as the main driving force for the clustering. This study offers a novel outlook on future investigations of stability and degradation reaction mechanisms of nanocatalysts in electrochemical CO_2_ reduction and, more generally, in electroreduction reactions.

## Abstract (web summary)

While the degradation of materials during usage is crucial in understanding their performance, it is challenging to understand the corrosion processes. Here, authors find copper nanoparticles to undergo an unusual potential-driven nanoclustering degradation pathway during carbon dioxide reduction.

## Introduction

Correlating activity, selectivity, and stability with the morphology and composition of nanocatalysts is crucial to advancing the knowledge in chemical transformations which are important to progress toward a more sustainable economy. Yet, still more emphasis needs to be placed on elucidating catalyst degradation mechanisms^1^. Electrochemical CO_2_ reduction reaction (CO_2_RR) into value-added chemicals is among the most challenging, underexplored and fast developing fields in catalysis. Because it enables concurrent carbon fixation and renewable energy storage, this conversion represents a sustainable approach to mitigating global challenges arising from the substantial utilization of fossil fuels and from the ever-increasing atmospheric CO_2_^2^. Cu is an appealing catalyst for CO_2_RR, as it produces appreciable amounts of highly sought after energy-dense hydrocarbons, such as CH_4_, C_2_H_4_, and C_2_H_6_^3–15^. Among different catalysts, Cu nanocubes (CuNCs), synthesized by various approaches, were found to exhibit exceptionally high selectivity for C–C bond formation^13–15^, with this selectivity being size-dependent^14^. Bi-functionality of {110} edge- and {100} plane- atoms was suggested as the underlying reason of such behavior^14^. As mentioned, from the perspective of practical implementation, long-term stability is a key parameter beyond activity and selectivity for real-world catalysts^1,16,17^. Nevertheless, the stability of CO_2_ reduction catalysts and their degradation mechanism during extended electrolysis has not yet been sufficiently investigated^18–20^.

Here we report the discovery of a potential-induced nanoclustering degradation mechanism in CuNCs during extended electrochemical CO_2_RR. We investigate the structural evolution of CuNCs with three distinct sizes (i.e., 16 nm, 41 nm, and 65 nm) to correlate with their activity, selectivity and stability. Our results establish that the three sized CuNCs feature uncommon nanoclustering followed by a coalescence process. The state-of-the-art synthesis and characterization are complemented with theoretical investigations which highlight the role of the applied negative potential as the main driving force for the loss of the pristine cubic morphology. Our insights into the degradation mechanism of Cu nanocatalysts offer a unique outlook to study the stability of newly developed CO_2_RR electrocatalysts and open the way towards defining mitigation strategies to improve this stability.

## Results

### Morphological evolution during electrolysis

**Fig. 1:**
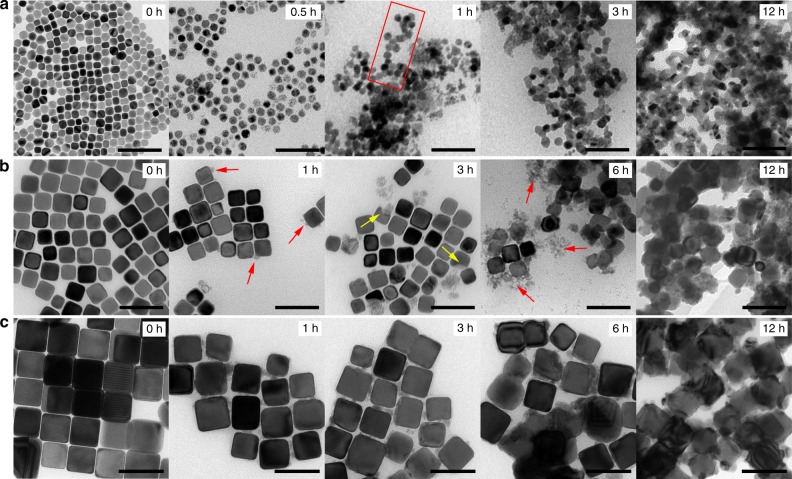
Morphological evolution of the CuNCs during electrolysis. **a**–**c** Representative CuNCs of three different sizes: **a** 16 nm, **b** 41 nm, and **c** 65 nm, imaged with TEM at different operation times. The rectangle in **a** encloses an aggregated assembly of particles. The red and yellow arrows in **b** indicate small clusters and broken CuNCs, respectively. Scale bars: 100 nm

The CuNCs were synthesized by a colloidal chemistry approach (Fig. 1 and Supplementary Fig. 1). This technique was chosen because it allows the exquisite size monodispersity and shape uniformity which is required to unambiguously identify degradation pathways through morphological evolution studies, while possessing the potential to close the gap between fundamental studies and technological implementation thanks to the possibility for scale-up. To investigate their stability, the NCs dispersed in hexane were loaded onto glassy carbon substrates and subjected to electrolysis in 0.1 M KHCO_3_ at −1.1 V vs. reversible hydrogen electrode (RHE) up to 12 h. The reaction was stopped at different times and the reacted NCs were then collected for transmission electron microscopy (TEM) analysis (see Methods for details). Figure 1a shows typical TEM micrographs of the pristine and reacted 16 nm CuNCs. Strikingly, while cubic contours are still discernable in the first 0.5 h, most CuNCs evolve into smaller and rounded particles due to a rapid detachment of nanoclusters (~3 nm) from the NCs. As the reaction proceeds for 1 h, both the particles and nanoclusters appear to assemble randomly and as a result different sizes and morphologies of aggregates are formed. Some aggregates of small particles exhibit a dendritic morphology, which is usually a result of random collision of particles^21^. Aggregate formation becomes predominant in the samples examined at the reaction times longer than 3 h. Here, the beads, i.e. the constituents of the aggregates, coalesce with each other to some degree, as is reflected by the larger necking areas of two neighboring ones. Eventually, when the reaction is prolonged to 12 h, the boundaries between beads are no longer clearly seen. Instead, all beads fuse together and tangled and slightly thicker networks are formed. The thicker network is probably caused by the coalescence of nanoclusters together with the beads.  When turning to the 41 nm CuNCs, they exhibit a similar pathway but a different evolution rate (Fig. 1b). In the 1 h sample, only a few CuNCs break into nanoclusters. As the reaction unfolds, nanoclustering becomes slightly intensified, which is reflected by several broken cubes in addition to lumps of clusters in the vicinity (Fig. 1b, 3 h sample). Although, the longer reaction time aggravates the degradation, some pristine CuNCs are still observable after the reaction up to 6 h. At this moment, the degraded cubes are also seen to start to coalesce with each other, for which the clusters seem to serve as a binder. In the 12 h sample, all NCs are glued together. Interestingly, 65 nm CuNCs resemble 41 nm CuNCs, exhibiting similar morphological features at various stages, yet such changes are slightly delayed in time (Fig. 1c).

### Characterization of the nanoclusters

**Fig. 2:**
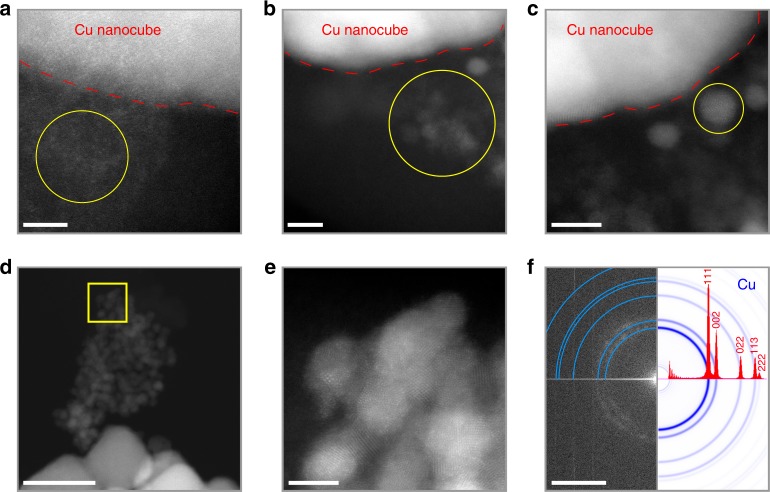
Characterizations of the Cu nanoclusters formed during electrolysis. **a**–**e** High-resolution HAADF-STEM images of **a**, **b** Cu nanoclusters (<3 nm) and **c**–**e** Cu nanoparticles (~5 nm) formed from the 41 nm CuNCs during electrolysis for 4.5 h under CO_2_RR conditions. Circles in **a**, **b**, and **c** enclose regions containing nanoclusters and nanoparticles, respectively. **e** is a high-magnification view of the region boxed in **d**. **f** FFT of the HR-STEM image shown in **e**. Simulated electron diffraction pattern (blue rings: ring sampling diffraction planes; red spectrum: intensity profile) of Cu are included for reference. Scale bars: **a** 3 nm, **b**, **c**, **e** 5 nm, **d** 50 nm, and **f** 5 nm^−1^

To gain more insights into the nanoclustering phenomena, we performed detailed aberration-corrected high-resolution TEM characterizations on the clusters (<3 nm) and particles (~5 nm) which form from the coalescence of the former. Figure 2 reports the high-angle annular dark-field scanning TEM (HAADF-STEM) characterization results. As it can be seen from Fig. 2a, when the clusters consist of only a few atoms, they are amorphous. Because of their high mobility, some clusters coalesce into larger particles of ~2–3 nm (Fig. 2b) and of ~5 nm (Fig. 2c–e). The former show a certain degree of short-range ordering while the latter display clear crystalline fringes that contain useful structural information. The fast Fourier transform (FFT) pattern reveals that these particles are pure metallic Cu (Fig. 2f). Selected area electron diffraction (SAED) pattern in TEM mode was further acquired on a more extended area (Supplementary Fig. 2). Interestingly, very weak reflections corresponding to Cu_2_O were detected; however, this trivial amount of Cu_2_O was attributed to the native oxidation during the TEM sample preparation. Based on these results and analysis, it is concluded that the clusters are metallic Cu clusters. Advancing one step, one Cu particles of ~5 nm that contains penta-twins was captured (Supplementary Fig. 2c, f). This observation suggests that the ~5 nm particles form from the coalescence of smaller clusters, as penta-twinned particles cannot ‘detach’ directly from the single-crystalline CuNCs.

### Impact of the morphological changes on the CO_2_RR performance

**Fig. 3:**
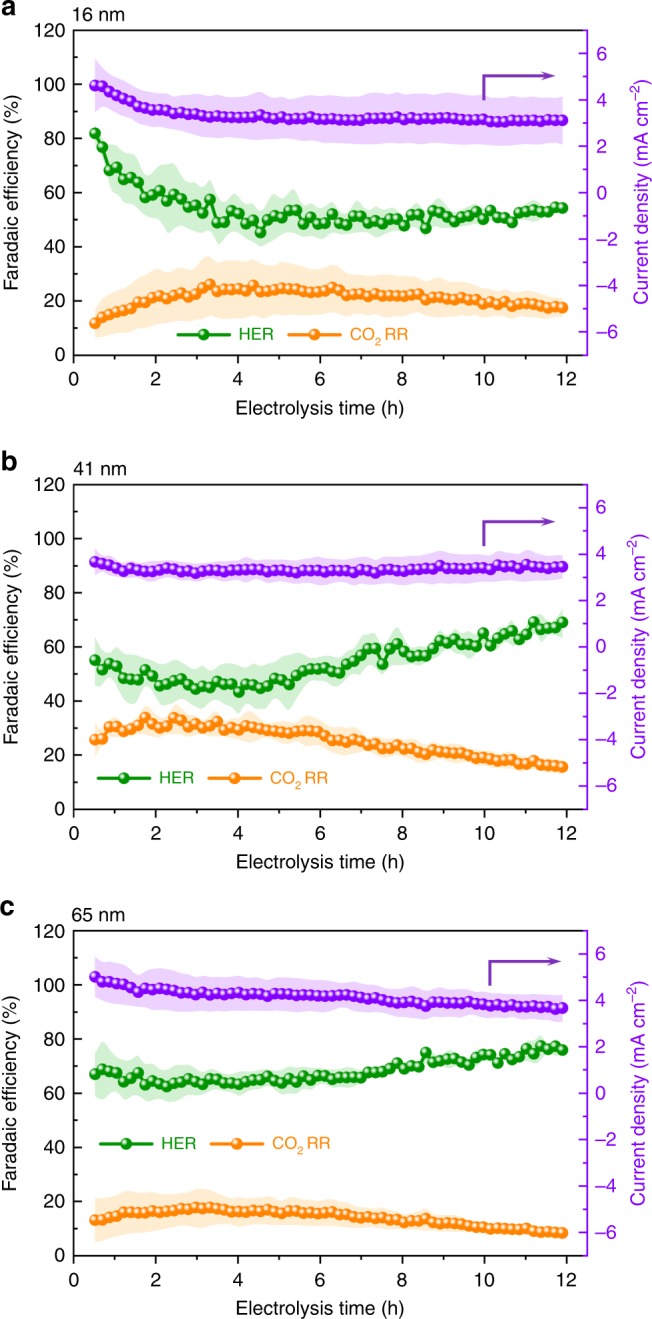
Electrocatalytic performance over time of the CuNCs. **a**–**c** Faradaic efficiency of gaseous products and current density from CuNCs of three different sizes: **a** 16 nm, **b** 41 nm, **c** 65 nm, collected during a 12 h-course of CO_2_RR. Shaded areas of each line show standard deviations from three independent measurements

To characterize the impact of structural changes on the electrocatalytic performance, we recorded current density and faradaic efficiency (FE) of CO_2_RR and of the competing hydrogen evolution reaction (HER), which are two descriptors used for electrocatalytic activity and selectivity, respectively (Fig. 3). The gaseous product distribution for CO_2_RR is reported in Supplementary Fig. 3. The general trend consisted in an increase of the FE towards HER as the nanocluster population increases, in agreement with previous findings that smaller Cu nanoparticles and nanoclusters with higher number of low-coordination sites favor HER at the expenses of the CO_2_RR^12,14,22^. For the 16 nm CuNCs, the intrinsic higher reactivity of such smaller particles and of the clusters, which form from nanoclustering at the beginning of the reaction (within the first 0.5 h, Fig. 1a), resulted in a high starting FE toward HER (0.5 h, Fig. 3a). As the aggregative assembly proceeded in the following 3 h, the current density decreased and the selectivity shifted from HER to CO_2_RR due to the loss of low-coordinated sites that are active for HER, which is reflected by the progressive decrease in hydrogen evolution accompanied by a steady increase of gaseous products of CO_2_RR (0.5–3 h, Fig. 3a). The further smoothening of the aggregates in the next 9 h only led to gradually favoring HER over CO_2_RR (3–12 h, Fig. 3a). Turning to the 41 nm and 65 nm CuNCs, these share similar trends in the FE of HER and CO_2_RR following their structural evolution: the FEs are stable for the first ~6 h until when HER starts to rise and CO_2_RR to drop, which happens at a slightly lower rate for the 65 nm compared with the 41 nm (Fig. 3b, c).The plateaus present for both HER and CO_2_RR indicate the higher structural stability of these bigger sized cubes compared with the 16 nm CuNCs, as the effects of nanoclustering are observed at later reaction times. The effect of nanoclustering and coalescence, both detrimental for CO_2_RR, becomes evident only after the initial ~6 h, which shifts the selectivity from CO_2_RR toward HER (6–12 h, Fig. 3b, c). At such a negative potential of −1.1 V vs RHE, the kinetically more favorable HER occurs readily even in the absence of a good catalyst, thus it is not impacted by coalescence as much as CO_2_RR. The stability of the total current density during the 12 h likely derives from a compensating effect between the increasing HER partial current density and the decreasing CO_2_RR partial current density. Taken together, these results suggest a strong correlation between the structural evolution and the electrochemical behavior during CO_2_RR.

It should be noted that the combination of X-Ray diffraction (XRD), X-ray photoelectron spectroscopy (XPS) and Auger spectroscopy (Supplementary Fig. 4) suggests that the CuNCs are only lightly surface oxidized before electrochemistry. Based on recent reports in the literature using O^18^ labeling techniques and in situ Raman, the native surface oxide is expected to get fully reduced upon the application of the negative potential^23,24^. While the impact of this change of chemical state on the surface reconstruction cannot be fully excluded, considering the very low surface oxidation of the CuNCs when compared with O_2_ plasma treated copper^9^, it is concluded that it has a negligible influence on both the nanoclustering and electrocatalytic performance (Supplementary Fig. 4). Moreover, liquid products were also produced, as reported in Supplementary Fig. 5. Except for formate whose FE is ~10% in the first 1 h, all the other detectable liquid products (i.e. acetate, ethylene glycol, ethanol, 1-propanol) correspond to a FE below 5%. The non-trivial FE of formate in the first 1 h is likely due to the initial surface oxide which has been reported to facilitate the formation of formate^25^. In addition, ligands are generally believed to lower the activity of catalysts, because they might block active sites, as well as impede electron injection. Herein, Fourier transform infrared spectroscopy (FTIR) and XPS (Supplementary Fig. 6) evidence that the native ligands are stripped off by the very negative potential within the first 1 h of electrolysis, which is consistent with previous findings^21^. Therefore, the impact of the ligands on the electrochemical performance, if any, is expected to take place only at the beginning of the reaction. As a control experiment, ligand-stripped CuNCs were tested (Supplementary Fig. 7). The only difference compared with the ligand-functionalized CuNCs was a steady value versus a gradual increase of CO_2_RR FE during the first 1 h of electrolysis (cf. Supplementary Fig. 7 and Fig. 3b). The current was constant in both cases and the FE trends were nearly identical in the following few hours, thus confirming that indeed the native ligands impact the electrochemical performance only at the beginning of the measurements. While the deposition of graphitic carbon has been proposed as possible de-activation mechanism favoring HER over CO_2_RR^26,27^, this phenomenon has been observed when methane is the major reduction product^26,27^, which is not the case of this work (Supplementary Fig. 3). Furthermore, the evident correlation between structural changes and performance suggests that the deposition of graphitic carbon plays a minor role in our system, if any.

### Atomic-scale analysis of the structural degradation

**Fig. 4:**
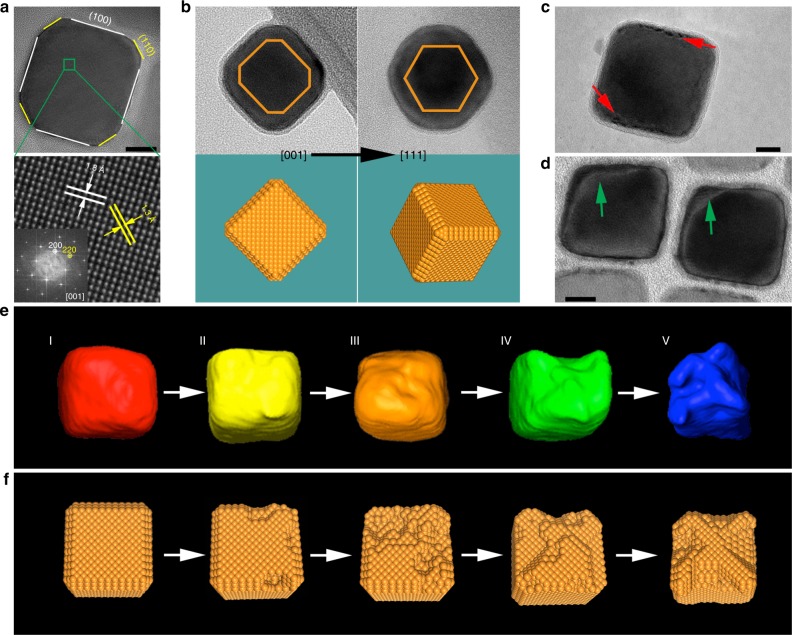
Crystalline facets and degradation pathway. **a** Top, HR-TEM image of one 41 nm CuNC along the [001] crystallographic direction; Bottom, an enlarged view of the region marked in the top panel. The inset shows the fast Fourier transformation (FFT) of the HR-TEM image. **b** TEM images and corresponding morphological models of one CuNC along two crystallographic directions, [001] and [111], from left to right. **c**, **d** TEM image of one CuNC electrolyzed for **c** 1 h and **d** 3 h. The arrows point to the positions where the degradations take place. **e** Tomographic reconstruction of the CuNCs and **f** corresponding schematic morphological models at different stages during a 12 h-course of CO_2_RR. Scale bars: 10 nm

Although a clear morphological evolution was observed from the low resolution TEM images (Fig. 1), high resolution TEM (HR-TEM) and electron tomography were key to unravel the degradation pathway of the CuNCs (Fig. 4 and Supplementary Figs 8, 9). The three sized CuNCs have identical major facets (Supplementary Fig. 10). The following analysis focuses on the 41 nm CuNCs because of their ideal ratio between facets that leads to a higher selectivity towards C–C bond formation (Supplementary Fig. 11)^14^. Firstly, the pristine CuNCs were analyzed by HR-TEM, and the CuNCs were imaged at two different crystallographic directions to determine the crystalline facets exposed on the surface (Fig. 4a, b). In addition to the six {100} facets, the edges were found to consist of {110} facets and small {111} corners. The latter, however, had limited effects on the electrochemical behavior, as it is evidenced by the low FE of CH_4_ whose formation is usually favored over Cu {111} (Supplementary Fig. 3)^28^. Figure 4c, Fig. 4d show the CuNCs that were electrolyzed for 1 h and 3 h, respectively, which clearly point out that degradation initiates from the edges of the cubes. The 2D projection nature of these images potentially hides some structural information that can be crucial for understanding the degradation mechanism. We therefore employed HAADF-STEM tomography, which is particularly advantageous for reconstructing 3D crystalline metallic nanostructures. Figure 4e shows the reconstructed tomographic volumes of the pristine and reacted CuNCs at different stages. The reconstructed pristine cube shows a slightly truncated shape (I in Fig. 4e), which matches well the fact that the major facets of the cube are {100} and {110} facets. At the early stage of the electrolysis, the CuNC went through a pitting process, leaving tiny pinholes at edges of the cube (II in Fig. 4e), due to the removal of Cu in the form of nanoclusters as reveled in Fig. 1b. Over time the pinhole at the {100}/{110} interface enlarged its dimensions within the cube by propagating into an adjacent {100} plane and creating a rough surface with more pinholes (III in Fig. 4e). These new pinholes serve as new pitting centers, making the degradation running at multiple spots in parallel (IV in Fig. 4e). As each pitting continues, an irregularly shaped cubic framework with deeper pinholes eventually forms (V in Fig. 4e). A schematic illustration of the initiation, propagation and deepening of the pitting process which results in the nanoclustering is shown in Fig. 4f. Throughout the whole-pitting progress, the edge {110}—to—plane {100} ratio, which accounts for the selectivity toward ethylene^14^, changes, thus impacting the ethylene FE (Supplementary Fig. 3). The methane FE follows a similar trend of the ethylene FE over time. Nevertheless, linking the selectivity toward methane to the observed structural changes is not trivial due to the small fraction of Cu {111} in the pristine cubes.

### Investigation of the parameters driving degradation

It is well known that nanoparticles, because of their highsurface-to-volume ratio, tend to undergo sintering into larger particles to lower the overall surface energy. Ostwald ripening, reprecipitation and coalescence are the most common degradation mechanisms for nanocatalysts^1^. The first two follow from the catalyst dissolution, as ions go into solution, and then redeposit; the latter requires diffusion of the entire particles. Coalescence of particles into aggregated assembly was eventually observed in the three sized CuNCs and it was always sequential to the nanoclustering process; but it started at different times (after 0.5 h for the 16 nm NCs and after 6 h for the 41 nm and the 65 nm NCs, Fig. 1). Nanoclustering seems to be universal for Cu metal catalysts: it was also observed in 8 nm Cu spheres and 135 nm Cu octahedra by us (Supplementary Fig. 12), and in several-micrometer ultrathin Cu nanowires^11^ and copper thin films^18^ by other groups. Dissolution/reprecipitation reaction of the CuNCs could explain the formation of the nanoclusters. However, since the potential applied throughout CO_2_RR is always below the oxidation potential of the CuNCs, which we measured to be around 0.75 V vs RHE under the CO_2_RR conditions, dissolution of the CuNCs as Cu ions in solution is unlikely. As a matter of fact, the concentration of Cu ions in the electrolyte before and after electrolysis was within the error range of the quantitative elemental analysis conducted by inductively coupled plasma optical emission spectroscopy. Taking into account that nanoclustering is uncommon for nanoparticles dispersed on solid substrate at room temperature, due to the insufficient thermal energy (~25.7 meV) provided to surpass the cohesive energy of Cu (3.49 eV)^29^, we believe that the general occurrence of nanoclustering observed for Cu nanocrystals must originate from the CO_2_RR conditions.

In order to understand the influence of the CO_2_RR operation conditions on nanoclustering, we examined the effects of the sole CO_2_ adsorption and of the negative potential by cutting off the voltage supply and the CO_2_ flow, respectively, while keeping all other conditions identical. Interestingly, significant nanoclustering was observed when the negative voltage was applied, even in the absence of the CO_2_ flow (Supplementary Fig. 13); thus, these experiments suggested a major role played by the voltage in the degradation of the CuNCs. While nanoclustering has not been studied in nanoparticle electrocatalysts, one recent study on bulk Cu surfaces suggests that the mechanism behind the contribution of CO_2_ to the nanoclustering may involve the dissociation of CO_2_ on Cu nanocrystals which releases energy exceeding that required for the breaking of Cu–Cu bonds and consequently leads to the breaking up of the Cu surface into nanoclusters^30^. On the other hand, it is unexpected to see that such nanoclustering in metallic catalysts is mainly driven by the negative potential. While reduction of the ligands and consequent stripping from the CuNCs surface is induced by the negative potential during electrolysis (Supplementary Fig. 6), we verified that the sole ligand removal is not cause of the degradation; in fact, intact CuNCs and no nanoclustering were observed when the ligands were intentionally removed from the surface by plasma treatment. The impact of ligand removal was mostly on the degradation kinetics during CO_2_RR as faster morphological and performance changes were observed in the bare CuNCs compared with the pristine ones (Supplementary Fig. 14). Therefore, the negative potential must play other role(s), most likely to do with the stability of the nanocrystals themselves.

**Fig. 5:**
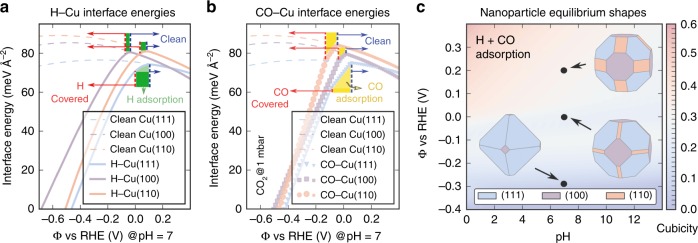
Theoretical DFT investigations. **a**, **b** Grand potential interface energies for **a** H-covered and **b** CO-covered Cu surfaces in aqueous solution. **c** pH and potential dependence of the Wulff-shape of Cu nanoparticles including H, CO, and mixed H + CO covered surfaces. Please note that the inaccuracy of our potential scale is around 0.3 V (see Supplementary Methods for details). The cubicity is defined as the relative contribution of Cu (100) surfaces to the Wulff-shape

In order to gain insight into the driving forces behind the CuNCs degradation under operation conditions, we performed density functional theory (DFT) calculations for bulk copper and (111), (100), and (110) surfaces within a grand canonical approach^31^, including also the (2 × 1) reconstructed (110) and the (5 × 1) reconstructed (100) surfaces (see Supplementary Methods). Clean, CO-covered, H-covered and mixed H + CO covered surfaces were examined. The CO molecule is an obvious choice to exemplify the C-containing intermediates during the conversion of CO_2_ to hydrocarbons because it serves as the common intermediate that precedes the formation of almost all the products (e.g., methane, ethylene, ethanol, etc.) and its coupling and/or protonation dictate the selectivity and efficiency of CO_2_RR^3^. At the same time, the omnipresence of HER under aqueous conditions, the participation of H- in the CO_2_RR proton-mediated steps, the stability of the CuH phase in the calculated Pourbaix diagram at the operating potential and pH (Supplementary Fig. 15) and the experimental evidence that HER induces nanoclustering as well (Supplementary Fig. 16) motivated us to consider the effect of proton electrosorption on Cu nanoparticle stability. pH- and potential-dependent interface energies were calculated for clean surfaces as well as for surfaces with different adsorbate configurations (0.25–2.0 monolayer coverages) using a slab approach, representing the water environment through the self-consistent polarizable continuum solvation model (SCCS)^32^. The methodology and all the detailed results are presented in Supplementary Methods. Herein, we focus the discussion on pH = 7, which is the experimental pH. The interfacial free energies (Supplementary Figs 17-19) and the surface terminations extracted from the Pourbaix diagrams (Supplementary Figs 15, 20) depend non-trivially on the pH and on the RHE scale. Nevertheless, unlike the studies by Kim et al. and Matsushima et al. that have found a cathodic polarization induced reconstruction of polycrystalline copper electrodes in either alkaline or acidic electrolytes^19,20^, at our operational potential we did not find a strong pH-dependence in the Pourbaix diagrams (Supplementary Fig. 15), which was further corroborated by the control experiments showing nanoclustering as a general phenomenon independent of the pH, and the choice and concentration of electrolytes (Supplementary Fig. 21). Therefore, the following discussion holds as well when assuming higher local pH due to the consumption of protons during the reaction. The interface free energies of (111), (100), and (110) Cu surfaces including the effects of H and CO adsorption are plotted in Fig. 5a, b, respectively. For clean surfaces, the interface energies at the potentials of zero charge (maxima of the dashed lines) are consistent with the well-documented thermodynamic stability *γ*_110_ > *γ*_100_ > *γ*_111_ and the reconstructed surfaces were found less stable than the unreconstructed ones (Supplementary Fig. 17 and Supplementary Table 1). In general, pristine bulk Cu surfaces are stable for potentials more positive than 0.1 V (blue arrows in Fig. 5a, b). More negative potentials induce H/CO adsorption (regions highlighted in green/yellow, and Supplementary Fig. 20), which in turn drives the electron transfer from the electrode toward the electrochemical interface built-up by the adsorbing species, and as a result, a strong reduction of interface free energies for fully covered surfaces (red arrows in Fig. 5a, b and Supplementary Fig. 19). The relative stability of the different facets changes for lower potentials in the presence of adsorbates to *γ*_100_ > *γ*_110_ > *γ*_111_ (Fig. 5a). For both adsorbates interface energies become negative between −0.4 and −0.5 V vs RHE @ pH = 7, except for H on Cu(100) (−0.68 V). Negative interface energies represent a strong driving force for CuNCs degradation by supporting the increase of absolute (111), (110), and (100) surface areas. In addition, for the pristine (110) surfaces, we found the unreconstructed surface more stable than the (2 × 1) reconstructed surface. Instead, upon hydrogen adsorption of up to 0.75 of one monolayer the reconstructed surface becomes clearly more stable due to strong adsorption at the step edges. This is in line with the general trend of higher reactivity of lower coordinated sites and the experimentally observed CuNC degradation starting from the edges. We also derived the potential- and pH-dependent Wulff-shapes from the interface free energies including H, CO and co-adsorbed H + CO terminations (Fig. 5c), we find that the cubicity of the polyhedron defined by (100), (110), and (111) facets under realistic CO_2_RR conditions decreases as the potential decreases. In other words, more negative potentials tend to stabilize non-cubic particles due to smaller interface energies for adsorbate-covered (111) and (110) Cu surfaces. The potential-driven degradation of the cubic morphology was corroborated by a control experiment in which a negative potential was directly applied to CuNCs deposited on a TEM grid (Supplementary Fig. 22). Furthermore, if it is true that the CuNCs undergo a potential-driven degradation, the CuNCs should be more stable at more positive voltages with other conditions unchanged. When the electrolysis was conducted at more positive voltages (−0.7 V and −0.3 V vs. RHE), the CuNCs indeed almost fully preserved their shapes and were isolated from each other (Supplementary Fig. 23), demonstrating the effectiveness of increasing potential as a mitigation strategy to impede nanoscale structural changes.

## Discussion

**Fig. 6:**
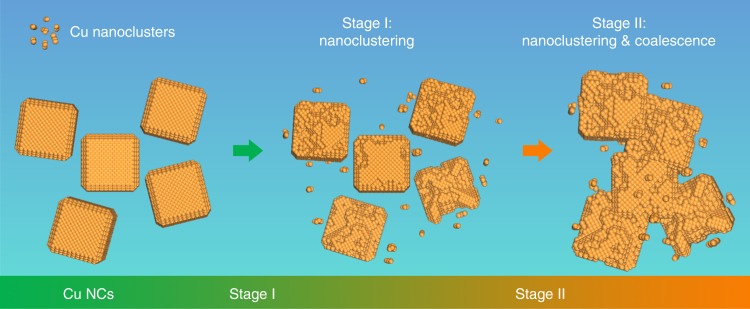
Overview of the degradation mechanism of CuNCs during CO_2_RR. Schematic representation of the degradation mechanism that includes nanoclustering (Stage I) followed by a coalescence at a later stage (Stage II)

The combination of electron microscopy characterization, product analysis and theoretical calculations provide the whole picture on the degradation mechanism of Cu nanocubes electrocatalysts during CO_2_RR, which is depicted in Fig. 6. The first stage is nanoclustering (Stage I). Our theory points out that the adsorption of either H- or CO- species on the crystal surface eventually induces degradation of the crystals under a sufficiently negative potential. Higher catalytic activity implies higher adsorbate coverage thus faster degradation. The 16 nm CuNCs are much more reactive and selective for HER than bigger particles, which accounts for the rapid nanoclustering observed in the 16 nm CuNCs. For the 41 nm CuNCs, the nanoclustering starts at the intersection between the {110} and the {100} facets, previously identified as the catalytic sites favoring CO_2_RR over HER^14^. This leads to the assumption that CO-adsorbates combined with the negative potential are driving the degradation. The newly formed surface of the pinholes forming from the nanoclustering event possesses a higher number of low-coordinated sites, which are more catalytically active. Thus, the pinhole rapidly enlarges its dimensions within the cube and creates a rough surface with even more pinholes. The 65 nm CuNCs exhibit a lower reactivity than the 16 nm CuNCs and a poorer selectivity towards CO_2_RR than the 41 nm CuNCs. This is likely the reason why they show a relatively slower nanoclustering process. As the nanoclustering continues, coalescence between the clusters and the degraded CuNCs starts (Stage II). The simultaneous phenomena of the reduction in size of the CuNCs, the increased cluster population and the ligand removal over time induce the formation of aggregated assemblies. A similar formation of aggregated assemblies during CO_2_RR had been already observed for 4 nm Au nanoparticles, which were also ligand-stripped during electrolysis^21^. These results indicate that the transport of entire particles more likely happens in small particles, which is reasonable considering that as the size drops the tendency to sintering to minimize surface energy and the mobility of the nanoparticles increase^33^. This explains the earlier temporal threshold for coalescence observed in the 16 nm NCs.

In summary, we have revealed an unusual nanoclustering degradation mechanism for nanoparticle electrocatalysts by monitoring the structural changes and catalytic behavior of three different sized CuNCs (16 nm, 41 nm, and 65 nm) over 12 h of electrolysis for CO_2_RR. The CO_2_ adsorption and the negative potentials were identified as the parameters accounting for the nanoclustering, with the latter outweighing the former. State-of-the-art grand canonical DFT calculations provided a confirmation of the role played by the negative potential as the main driving force of the observed morphological changes. By delivering a comprehensive understanding of the degradation mechanism, this study informs strategies for preserving Cu electrocatalyst nanostructures in order to maintain the electrochemical performance during CO_2_RR. From a mechanistic perspective, it motivates future research involving the introduction of suitable promoters (metal oxides, ligands, or another metal) that can reduce the overpotential of Cu electrocatalysts toward CO_2_RR through mutual interaction at the interface. From a practical perspective, wrapping the catalysts with a thin, porous, and conductive material, such as a graphene oxide^11^, can offer a good solution by physically impeding the detachment of clusters from the catalyst while still preserves the necessary conductivity and CO_2_ accessibility to the active sites. The extension of our study to different metallic nanoparticle electrocatalysts (e.g., Ag, Pd) proves the generality of the conclusions described here (Supplementary Fig. 24). Thus, our work opens new venues toward the understanding of the degradation reaction mechanisms of new electrocatalysts for CO_2_ reduction or other electroreduction reaction (e.g., nitrogen electroreduction), which will most likely differ from the more studied oxygen reduction and evolution catalysts^1,16,17,34^.

## Methods

### Synthesis of the CuNCs

CuNCs were synthesized following modified procedures introduced in our previous work^14^. Specifically, to synthesize 41 nm CuNCs, trioctylphosphine oxide (TOPO, 8 mmol) was first mixed with oleylamine (35 mL) in a three-necked flask under a magnetic stirring at room temperature (RT), followed by a pump vacuum for 20 min. CuBr (1.5 mmol) was then quickly added into the mixture under a protective atmosphere of N_2_, followed with a heating of the mixture at 80 °C for 15 min. After that, the resulting solution was rapidly heated up to 260 °C and kept refluxed at this temperature for 20 min before being cooled down to RT naturally. To synthesize 65 nm CuNCs, the same procedure for 41 nm CuNCs was followed, with the exception that the reflux time was prolonged to 2 h. The synthesis of 16 nm CuNCs also followed the similar procedure for 41 nm CuNCs, with two modifications: (1) the quantity of TOPO and oleylamine was increased to 12.5 mmol and reduced to 20 mL, respectively; (2) the reflux temperature and time were lowered and prolonged to 230 °C and 40 min, respectively. The obtained CuNCs were washed by adding a mixture of hexane and ethanol in the final reaction solution, followed with centrifugation at 5000 rpm for 5 min. Finally, the CuNCs were stored in toluene for further usage. Additional information of chemicals used in the synthesis, and synthesis of Ag nanocubes, Pd nanocubes, Cu octahedrons, and Cu nanospheres are reported in Supplementary Methods.

### Characterization methods

TEM images were acquired on FEI Tecnai-Spirit (at 120 kV) and Tecnai-Osiris (at 200 kV). HR-TEM, HR-STEM, and tomographic data were acquired on a FEI Titan Themis 60–300 operated at 300 kV. For tomography, a tilt-series of HAADF images (collection angle, 100–200 mrad) were collected over an angular range of −74° to +74° in 2° increments, using an incident beam of 10 mrad convergence semi-angle. The tilt-series images were aligned and a SIRT reconstruction (30 iterations) performed using FEI Inspect 3D software. The tomogram volume rendering and segmentation were done using Avizo software. XPS data were collected on a PHI VersaProbe II scanning XPS microprobe (Physical Instruments AG, Germany) with a monochromatic Al-Kα X-ray source operating at 24.8 W under ultrahigh vacuum conditions. XRD measurements were conducted on a BRUKER D8 Advance instrument with Cu Kα radiation. FTIR analysis was performed on PerkinElmer Spectrum Two.

### Electrocatalytic measurements

Electrocatalytic measurements were performed with a potentiostat (Biologic SP-300) in a custom-built gas-tight three-electrode cell. Typically, working electrodes were prepared by evaporating 15 µL hexane containing different amounts of CuNCs (16 nm: 22.6 µg, 41 nm: 14 µg, 65 nm: 38.6 µg) within a circular area of 1.39 ± 0.13 cm^2^ on the glassy carbon substrates. Here, the loading amounts were chosen to yield approximately the same current density for the three sized CuNCs and a uniform sub-monolayer of particles on the substrate, rather than to optimize the Faradaic efficiency (Supplementary Fig. 25). Before measurements, the electrodes were carefully flushed with ethanol and then dried with a N_2_ flow. The working electrodes were then held at a constant bias of −1.7 V vs. Ag/AgCl using chronoamperometry for a set time of up to 12 h. The potential was chosen based on our previous potential-dependent studies, revealing that at such negative potential the hydrogen evolution reaction is less favorable^14^. The solution resistance was determined and compensated using the in-built MIR function of the potentiostat, compensating for 85% of the resistance. During electrolysis, CO_2_ was constantly bubbled through the electrolyte at a flow rate of 5 sccm to prevent depletion of CO_2_ in the electrolyte and to allow continuous analysis of gaseous products via a GC. The flow rate of CO_2_ was controlled with a mass flow controller (Bronkhorst). During electrolysis, the CO_2_ with gaseous products was allowed to flow directly into the gas sampling loop of the GC for online gaseous product analysis, which took ~10.5 min for each run of analysis. After electrolysis, the liquid products were collected from both the cathode and the anode chamber and analyzed by the high-performance liquid chromatography (HPLC). More details on the measurements are provided in Supplementary Methods.

For TEM imaging, a TEM grid with a drop of hexane atop was placed on the glassy carbon to collect the reacted nanoparticles after electrolysis. Such a transfer of particles from glassy carbon substrates to TEM grids does not affect the particle shape and splitting, as it was demonstrated by a control experiment in which CuNCs transferred from the glassy carbon were found to be identical to those directly deposited on the TEM grid (Supplementary Fig. 26).

### Theoretical calculations

The thermodynamic stability of bulk Cu and Cu-water interfaces was analysed by determining the theoretical Pourbaix diagram and grand canonical interface energies from density functional theory calculations, using Quantum ESPRESSO^35^ with pseudopotentials from the SSSP library^36^. Interface free energies were determined using a constant Fermi level approach with the water environment represented by the self-consistent continuum solvation model (SCCS)^32^, as implemented in ENVIRON. A schematic of the computational setup and a summary of the considered Cu surfaces are present in Supplementary Fig. 27 and Supplementary Table 2, respectively. The detailed methodology is described in Supplementary Methods.

### Data availability

The authors declare that the main data supporting the findings of this study are available within the article and its Supplementary Information files. All DFT calculations were performed with the open source code ‘Quantum ESPRESSO’ and the module ‘ENVIRON’. All calculations were managed by the AiiDA python framework (www.aiida.net) which allows to manage and store all calculations and related results in an acyclic graph database enabling long-term storage of full data provenance. The authors declare that all relevant data are available from the authors upon reasonable request.

## Electronic supplementary material

Supplementary Information
