# Supplementary material for: Potential-induced nanoclustering of metallic catalysts during electrochemical CO2 reduction
Source: Nat Commun. 2018 Aug 6;9:3117. doi: 10.1038/s41467-018-05544-3 (PMC6079067; doi:10.1038/s41467-018-05544-3)
Supplement: Supplementary file 1 — Supplementary Information [file 41467_2018_5544_MOESM1_ESM.pdf]

Supplementary Information

Potential-induced Nanoclustering of Metallic Catalysts during Electrochemical CO_2 Reduction

Huang et al.

Supplementary Figures

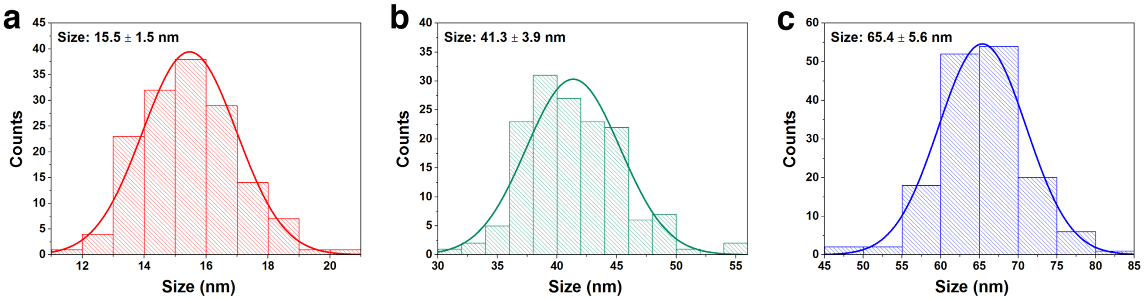

Supplementary Figure 1: Statistical analysis of the size of the CuNCs. **a-c** The histograms show that the cubes have a narrow size distribution and their sizes are around 16, 41 and 65 nm.

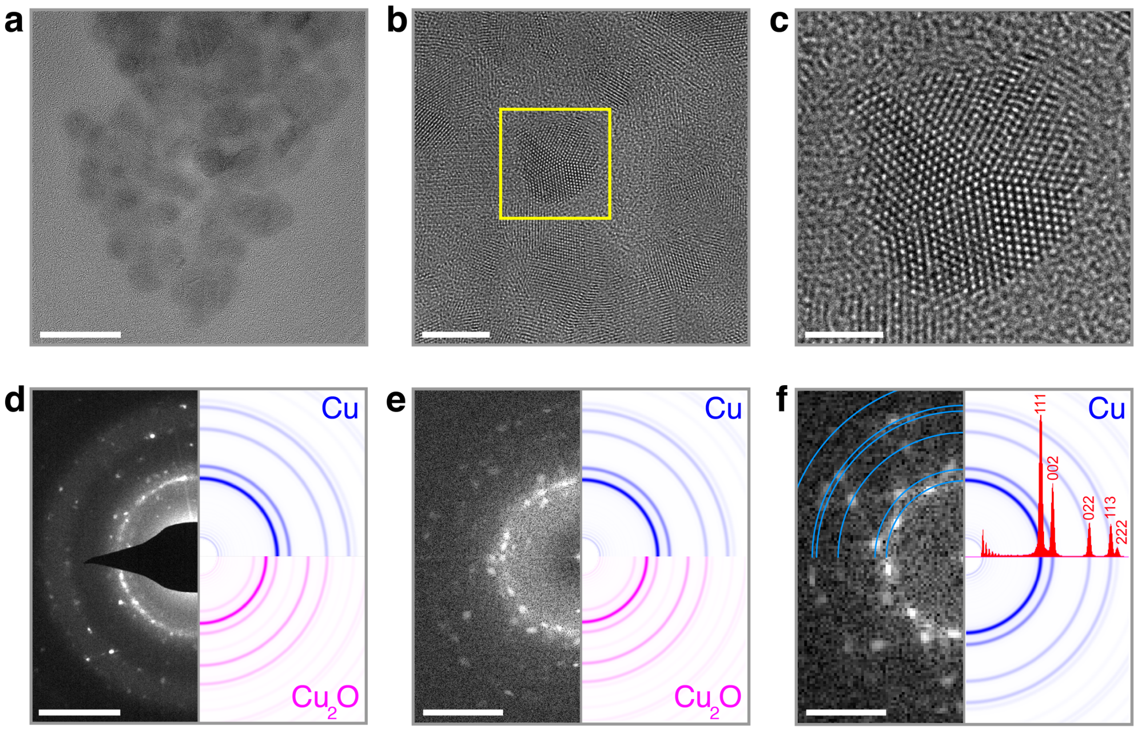

Supplementary Figure 2: Aberration-corrected HR-TEM characterizations of nanoparticles. **a-c** HR-TEM images, and corresponding **d** SAED and **e, f** FFT patterns of nanoparticles taken at progressively higher magnifications. The box in panel **b** encloses the particle shown in panel **c**. To the right of the SAED and FFT patterns are shown simulated electron diffraction patterns (blue and pink rings: ring sampling diffracting planes; red spectrum: intensity profile) of Cu and Cu_2O for reference. From **a, b, d, e** a barely-seen oxidation of Cu into Cu_2O was observed. In **c, f** one single penta-twinned Cu particles of $\approx$5 nm suggests that the particles form from the coalescence of smaller clusters, as penta-twinned cannot detach directly from the single-crystalline CuNCs. Scale bars: **a** 20 nm, **b** 5 nm, **c** 2 nm, and **d-f** 5 nm^{-1}

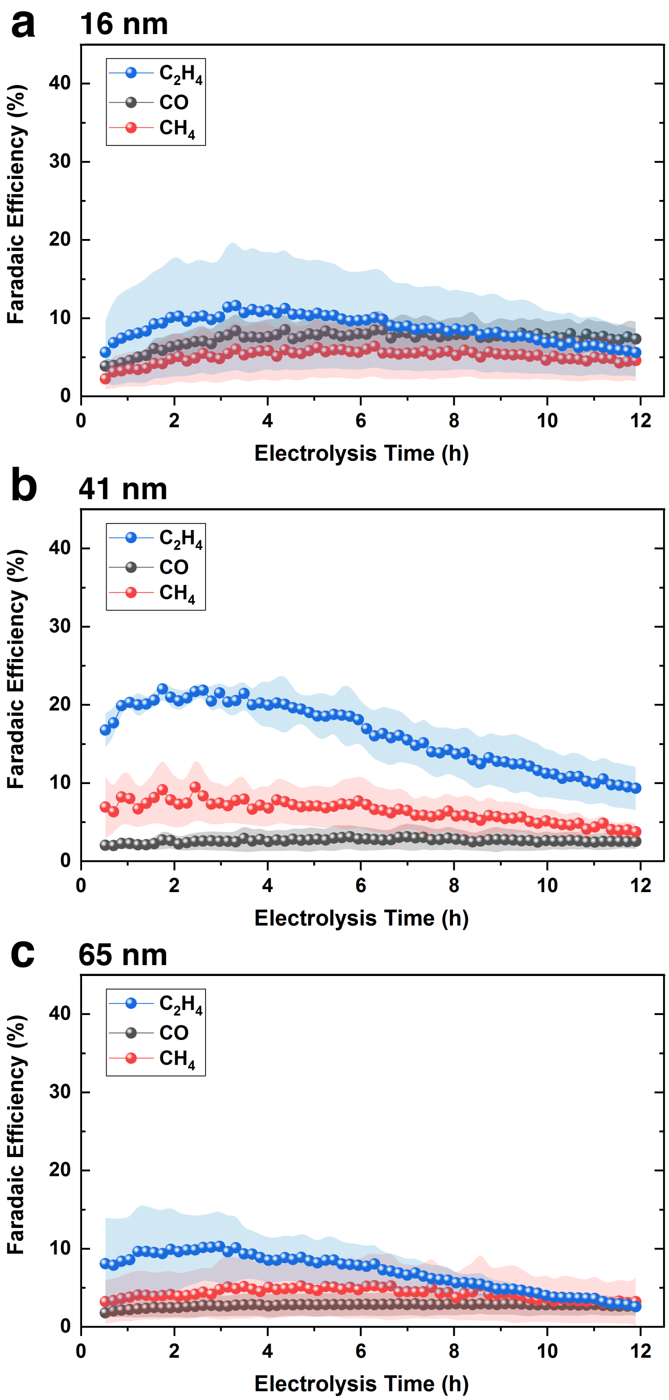

Supplementary Figure 3: Gaseous products of CO_2RR. **a-c** Faradaic efficiencies of gaseous products, i.e., C_2H_4, CH_4 and CO, for **a** 16, **b** 41 and **c** 65 nm CuNCs over a 12 h-course of CO_2RR. Shaded areas of each line show standard deviations from three independent measurements.

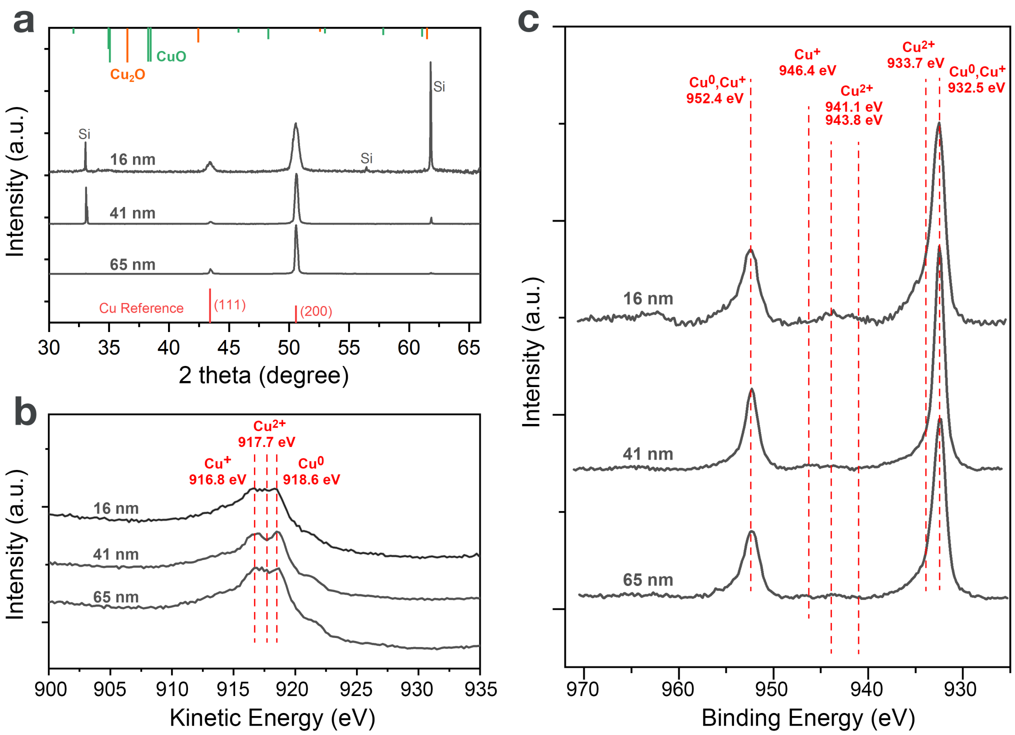

Supplementary Figure 4: Analysis of the oxidation state of the CuNCs. **a** XRD patterns showing a purely metallic Cu phase in all the three sizes. The Si peaks arise from the substrate used for these measurements. **b** Auger spectra evidencing the presence of the Cu^+. **c** XPS spectra signaling the possibility of some light surface oxidation. There have been some reports suggesting Cu^+ and the associated subsurface oxygen species to be the active site responsible for the selectivity of Cu-based electrodes towards C_2 and C_2^+ products [1, 2]. This hypothesis has been built by studying copper electrodes possessing a very thick copper oxide layer because derived from the reduction of copper oxide or because treated with oxygen plasma prior to electrochemistry. By contrast, the role of the native copper oxide which easily forms upon air exposure has been actually found to be insignificant [2]. In addition, it has been recently concluded by state-of-the-art in-situ Raman spectroscopy [3] and 18O isotope labelling [4] that the surface oxides even in the oxide-derived electrodes, are rapidly (within 10 min) and almost completely (< 1% residues) reduced under the high CO_2RR potential which is usually 1 V more negative than the standard reduction potential of Cu oxides. Our ex-situ data after electrochemistry were inconclusive due to the inevitable air exposure which causes the formation of the native oxide layer. Overall, in the present study, considering the insignificant amount of native oxide and the focus on long term stability, any major contribution of the Cu^+ species can be excluded.

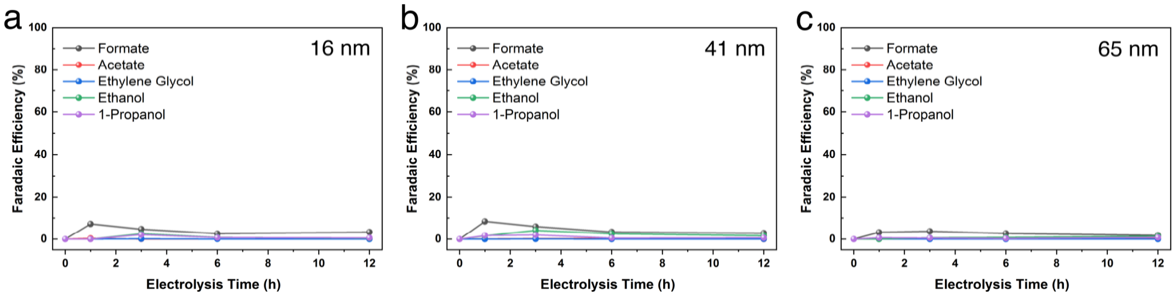

Supplementary Figure 5: Liquid product distribution over time. Time-averaged FE of liquid products for **a** 16 nm, **b** 41nm, and **c** 65 nm CuNCs.

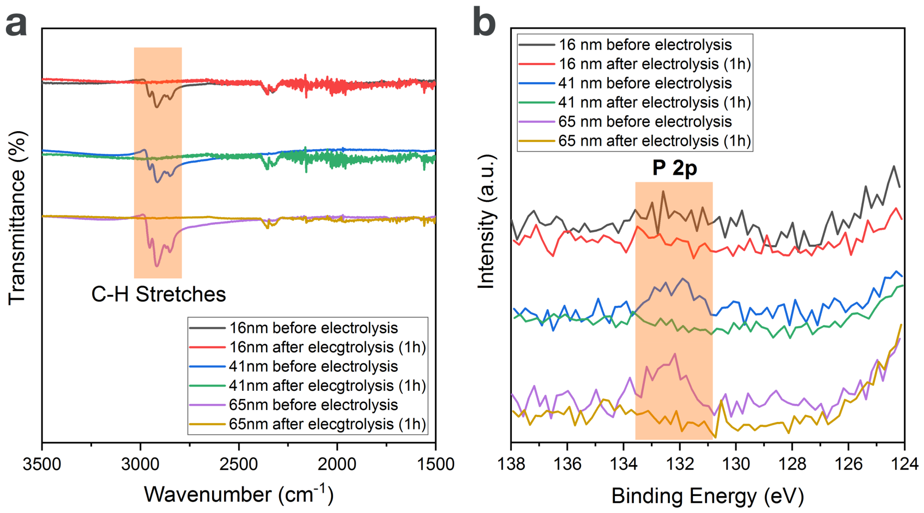

Supplementary Figure 6: Analysis of the ligands (i.e., trioctylphosphine oxide, TOPO) before and after electrolysis. **a** ATR-FTIR of the C-H bonds contained in the long TOPO C-chain and **b** XPS spectra of the P_{2p}, which is the characteristic element of TOPO, for 16 nm, 41 nm and 65 nm CuNCs before and after electrolysis under CO_2RR conditions for 1 h. The absence of C-H bonds and P_{2p} peaks suggests that the ligands have been stripped off after electrolysis.

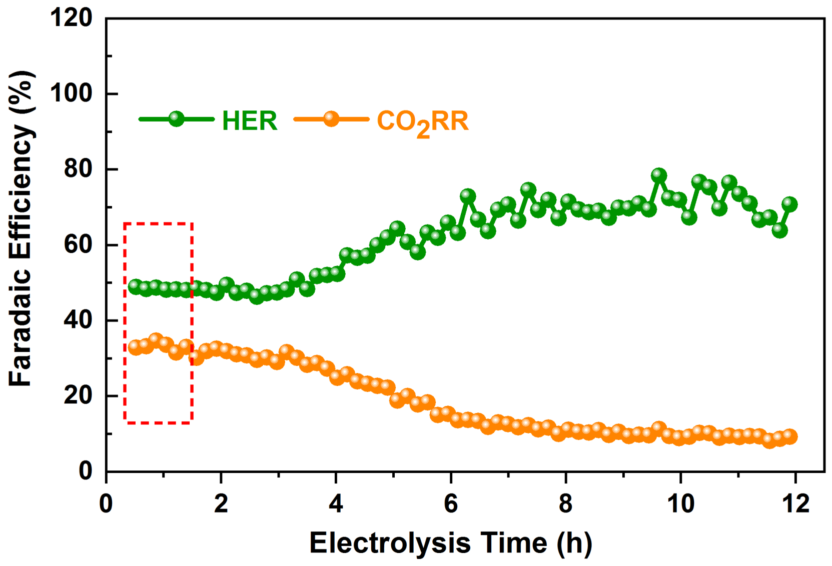

Supplementary Figure 7: Faradaic efficiencies over time of ligand-free CuNCs. Faradaic efficiency of N_2-plasma treated 41 nm CuNCs subjected to electrolysis under CO_2RR conditions for 12 h. The box highlights the constant FE in the first 1 h, suggesting a trivial involvement of the ligand in the electrolysis. Note: Plasma treatments were performed on a Femto low pressure plasma system (Diener electronic) with a power of 80 W.

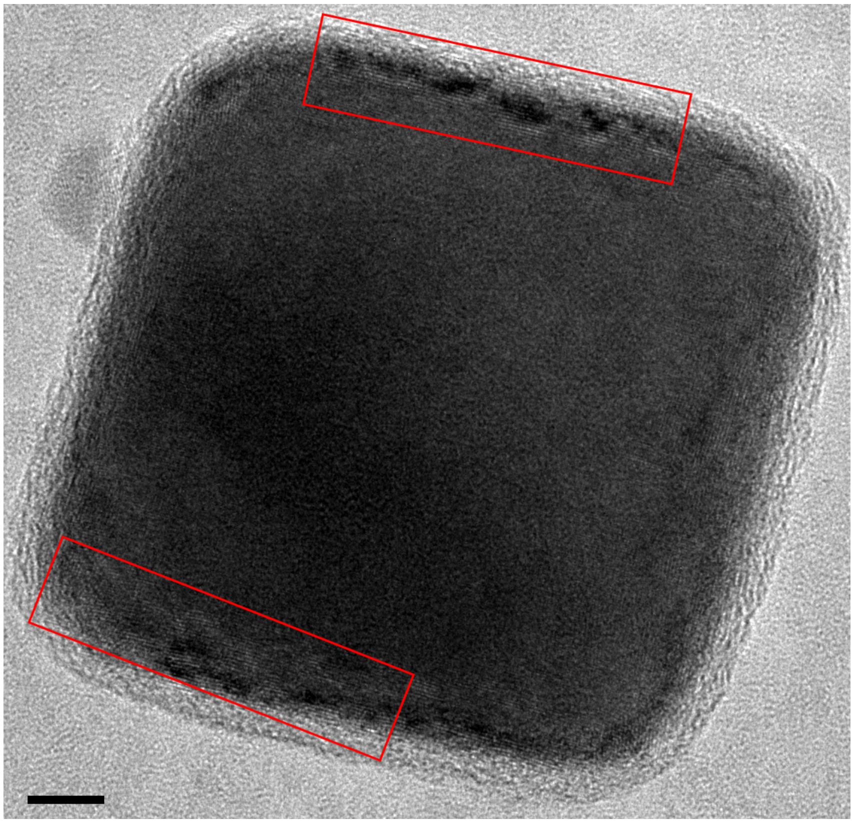

Supplementary Figure 8: Degradation at the atomic scale. HRTEM image of one CuNC that was electrolyzed for 1 h under typical CO_2RR conditions, confirming that the degradation starts from the edges of the cube. Scale bar: 5 nm.

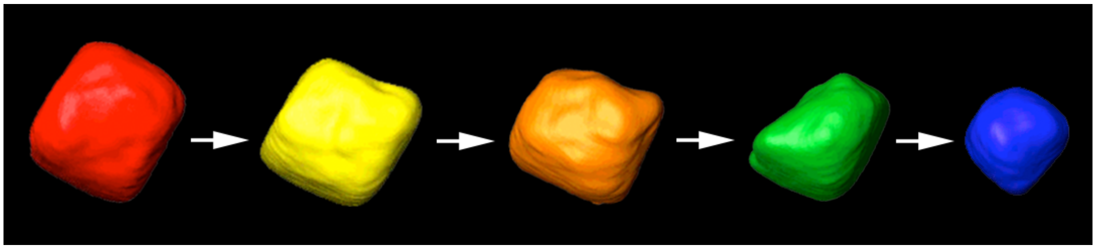

Supplementary Figure 9: Degradation in 3D. More HAADF-STEM tomography images of the Cu NCs at different stages during a 12 h-course of CO_2RR. Note: we examined a few more randomly selected CuNCs at each stages. Although the specific surface morphology of the reacted cubes varies a bit from those shown in Figure 4 **e**, the conclusion is consistent that the pitting process initiates from the edges, and then propagates to and deepens in the faces.

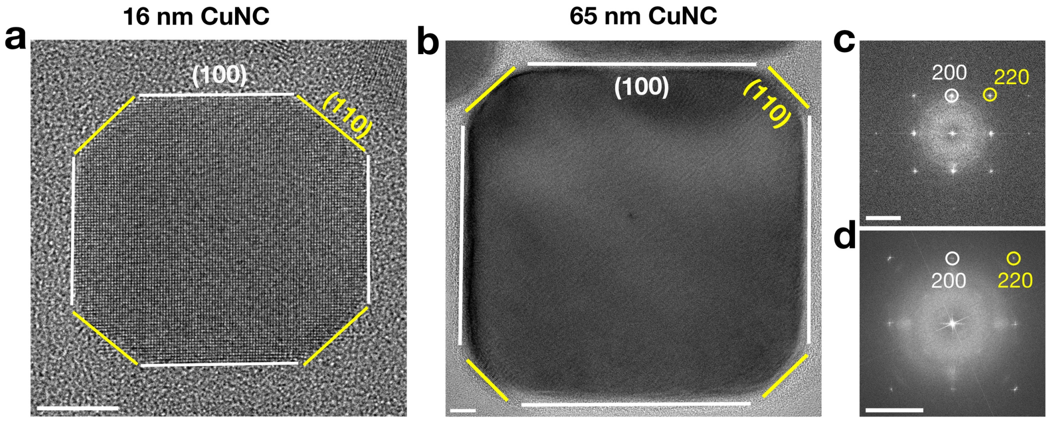

Supplementary Figure 10: High resolution TEM analysis of the 16 and 65 nm CuNCs. **a, b** Aberration-corrected HR-TEM images and **c, d** corresponding FFT patterns confirming that the $\{110\}$ and $\{100\}$ facets are the predominant facets for all the CuNCs. Scale bars: **a, b** 5 nm and **c, d** 5 nm^{-1}.

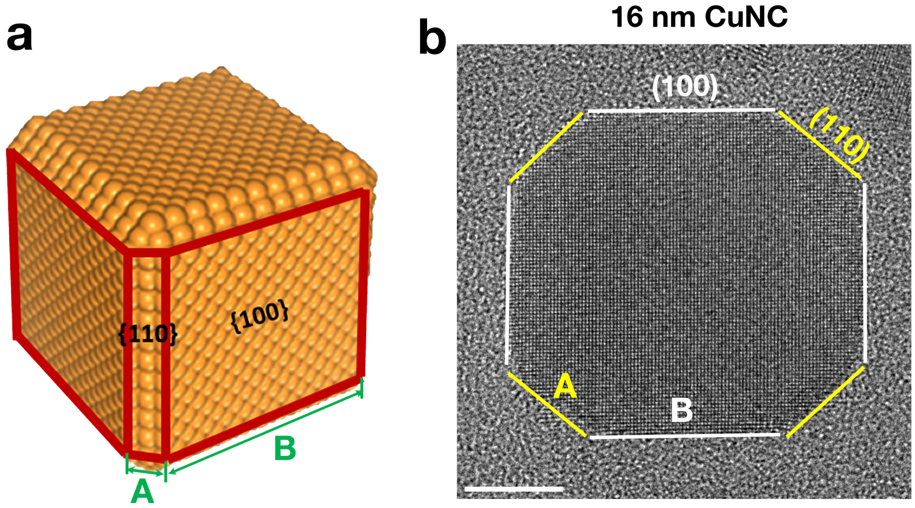

Supplementary Figure 11: $\{110\}/\{100\}$ ratio of Cu nanocubes. **a** schematic model of a cube and **b** HR-TEM image of a 16 nm CuNC showing edges of $\{110\}$ and $\{100\}$ facets. Scale bar: **b** 5 nm. Based on the schematic model shown in **a**, the $\{110\}/\{100\}$ ratio can be calculated in the following way:

$\{110\}/\{100\} = (12AB)/(6B^2) = 2A/B$

A and B can be easily measured from the edge length of $\{110\}$ and $\{100\}$ facets, respectively, in the 2D HR-TEM image **b**. Based on 30 particles of three distinct CuNCs sizes, we obtained the $\{110\}/\{100\}$ ratio as follows:

- $\{110\}/\{100\}(16\text{nm}) = 1.12 \pm 0.04$
- $\{110\}/\{100\}(41\text{nm}) = 0.75 \pm 0.08$
- $\{110\}/\{100\}(65\text{nm}) = 0.55 \pm 0.02$

Therefore, the CuNCs have size-dependent $\{110\}/\{100\}$ ratios.

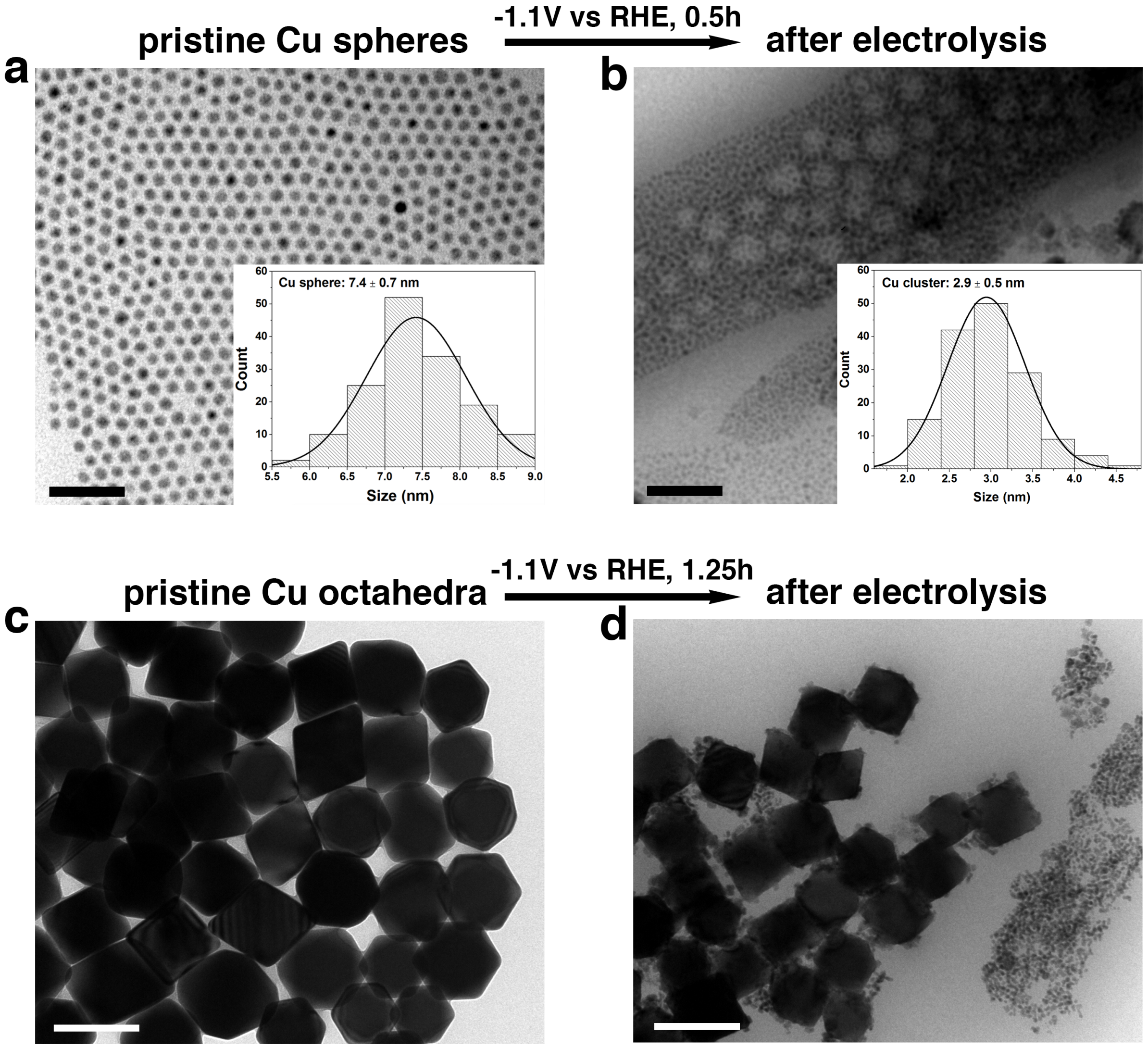

Supplementary Figure 12: Nanoclustering occurring in 7.4 nm Cu spheres and 135 nm Cu octahedra. TEM images of **a, c** pristine and **b, d** electrolyzed **a, b** Cu nanospheres and **c, d** octahedra, showing that nanoclusters were produced in spherical and octahedral shape of Cu-based electrocatalysts under CO_2RR conditions. Scale bars: **a, b** 50 nm and **c, d** 200 nm.

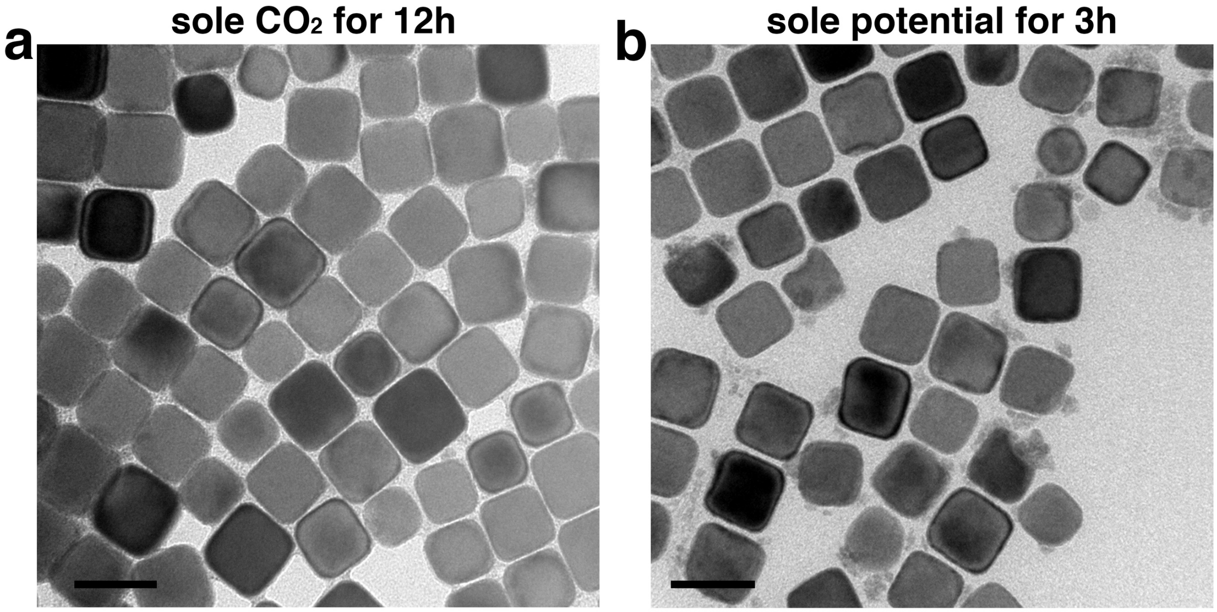

Supplementary Figure 13: The effect of CO_2 and negative potential on nanolcustering. **a, b** TEM images of 41 nm CuNCs that were collected from control experiments running for **a** 12 h and **b** 3 h, in which **a** negative potential and **b** CO_2 was cut off, respectively, and other conditions were identical to those typical for CO_2RR. In the control experiment designed for examining the effect of negative potential, the electrolyte (i.e., $KHCO_3$ solution) was bubbled with N_2 overnight to completely remove dissolved CO_2. Under the negative potential applied, therefore, only H_2 was produced. As can be seen from the two images, sole CO_2 adsorption has very trivial effect on inducing nanoclustering, while sole negative potential contributes majorly to the nanoclustering observed in Cu nanoparticles during CO_2RR. Scale bars: 50 nm.

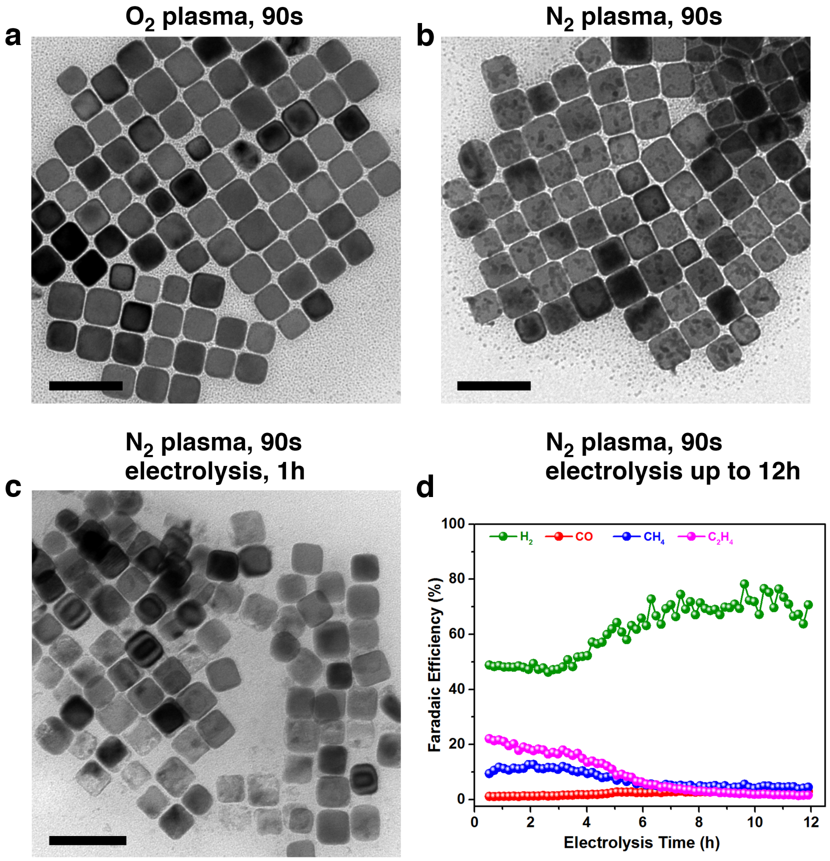

Supplementary Figure 14: Impact of the ligands on the degradation and electro-catalytic performance of 41 nm Cu NCs. **a, b** TEM images of CuNCs that were deposited on TEM Cu grids and subjected to **a** O_2 plasma and **b** N_2 plasma treatment, for 90 s. In both cases ligands are removed, yet nanoclustering is not observed. In the N_2 treated samples, carbon debris from decomposed ligands are observed; these carbon residues are present to a lower extent in O_2 treated samples due to the ligand desorption as CO_2. **c, d** TEM image and faradaic efficiency of N_2- treated CuNCs subjected to electrolysis under CO_2RR conditions for **c** 1 h and **d** 12 h. These data show that nanoclustering and degradation occur much faster than in the pristine CuNCs (cf. Figure 1 **b**). Accordingly, the decrease in selectivity for CO_2RR and the increase in selectivity for HER start at an earlier stage of the electrolysis (cf. Figure 3 **b**). For these experiments N_2-treated samples were chosen to exclude possible effects due to compositional changes, specifically surface oxidation. Note: Plasma treatments were performed on a Femto low pressure plasma system (Diener electronic) with a power of $\approx$ 80 W. Scale bars: 100 nm.

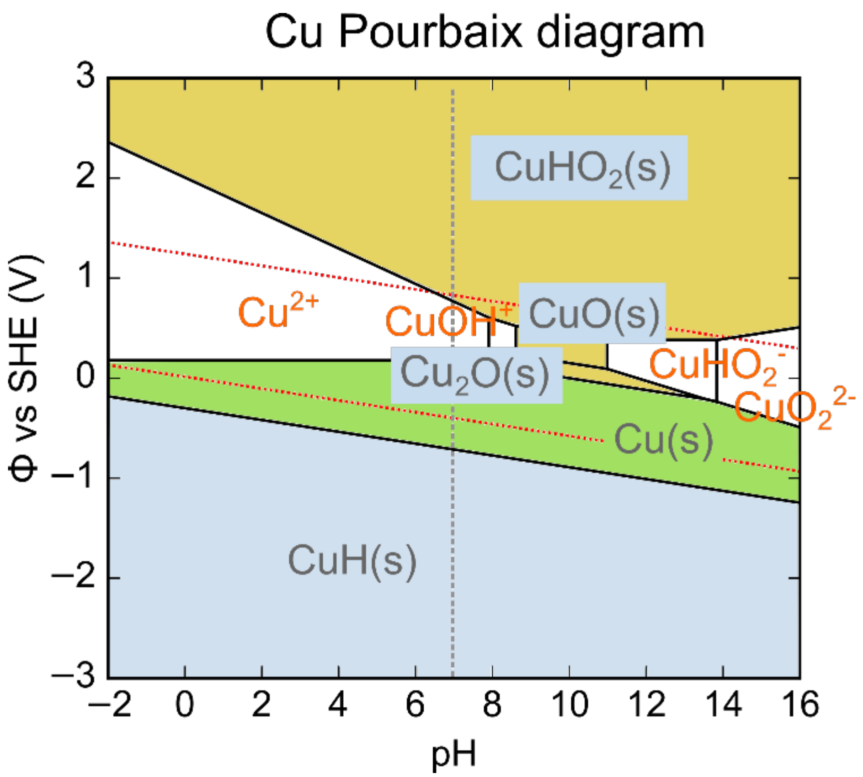

Supplementary Figure 15: Theoretically determined Pourbaix diagram of Cu in aqueous solution. The bulk stability of metallic copper can be analyzed from the theoretical Pourbaix diagram including all Cu, O and H containing compounds as available from the Materials Project database [5]. We found good agreement with the experimental Pourbaix diagram and the stability of metallic Cu in a potential window of approx. [-0.32 to +0.58 V vs RHE (pH 7)]. Whereas Cu gets oxidized for more positive potentials, CuH becomes a stable phase at potentials below -0.3 V, which indicates the presence of H covered surfaces and hydride surface phases. The negative potential, i.e. -1.1 V vs RHE, applied in this work, is more negative than any upper potential boundary of CuH(s) phase in the whole pH range, indicating the general validility of our discussion in the manuscript even when local pH is assumed to change due to the consumption of protons during the reaction.

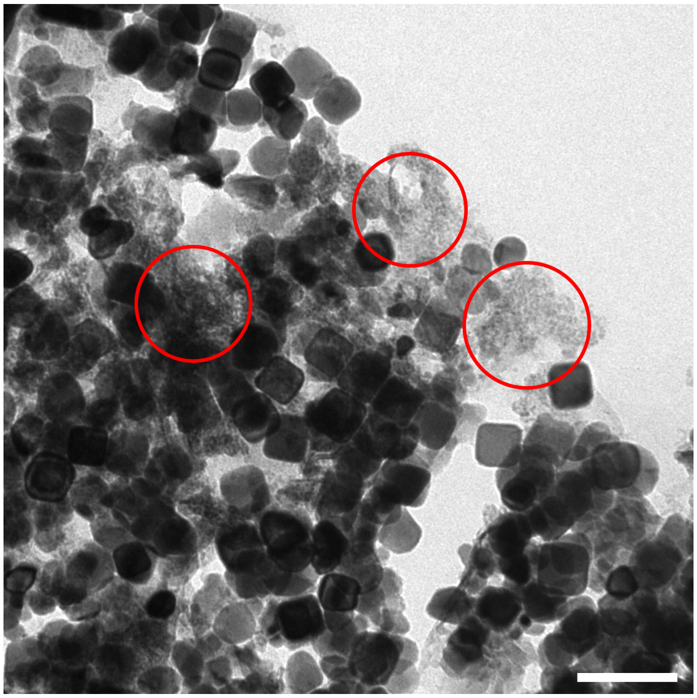

Supplementary Figure 16: Degradation of the CuNCs under HER conditions. TEM image of CuNCs that were electrolyzed at -1.1 V vs RHE for 3 h in 0.1 M $HClO_4$. No CO_2 flow was involved throughout the experiment. Gas chromatography detected H_2 as the only product. The circles enclose some clusters that formed during the electrolysis. Scale bar: 100 nm.

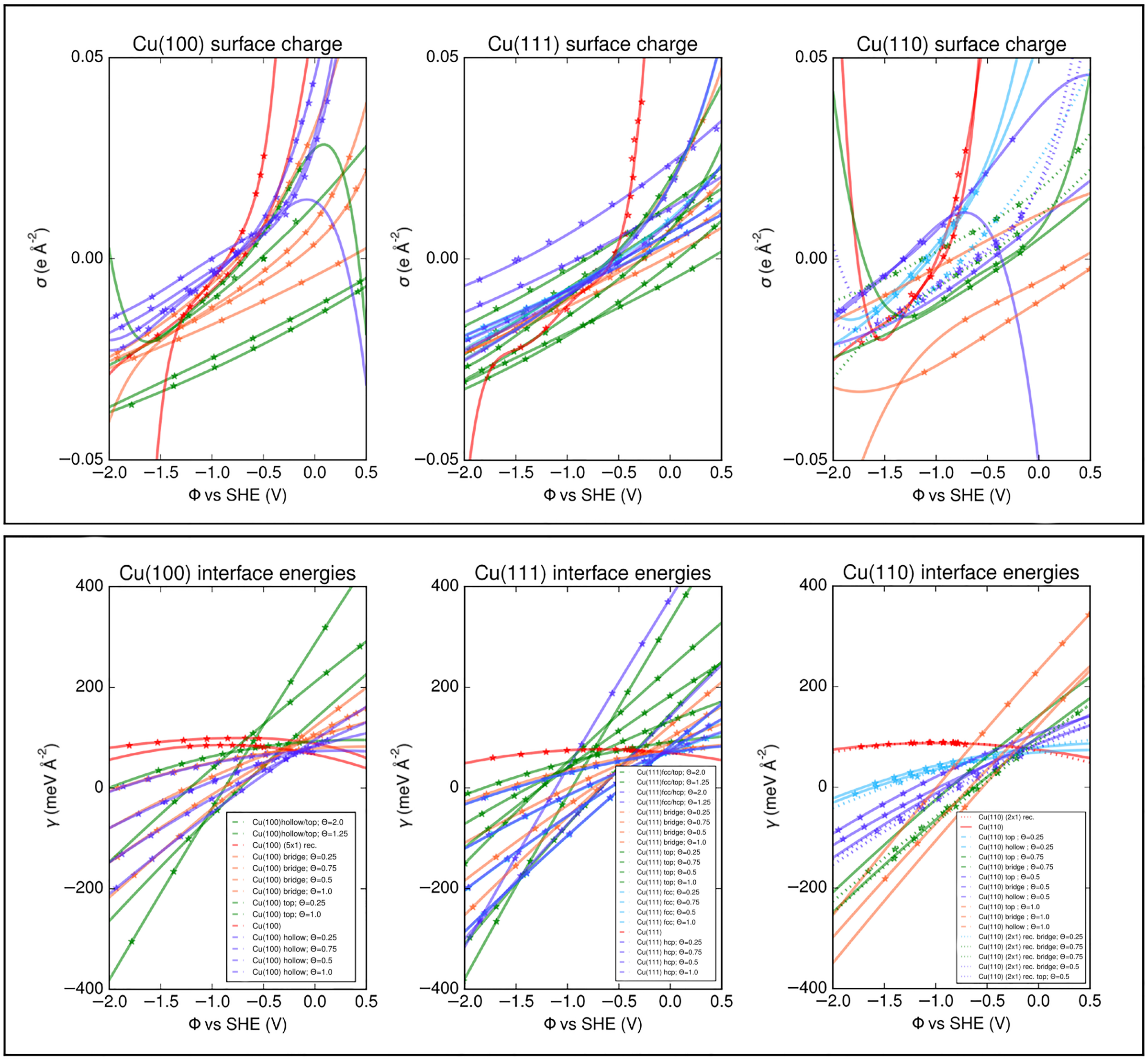

Supplementary Figure 17: Computational results for the charge-electrode potential dependence and interface energies. The stars represent the actually performed calculations. The upper subfigure plots the charge vs potential curve as obtained from the charge scans. The solid lines are polynomial fits, only used for the determination of interfacial capacitances at the PZC as tabulated in Supplementary Table 2. The lower subfigure plots the potential dependent interface energies as evalutated via Legendre transform with respect to charge of the charge dependent calculations, together with the second order potential fits for the interface energies, evaluated for pH = 0. The restriction to a second order polynomial in the fit does not limit the fit accuracy.

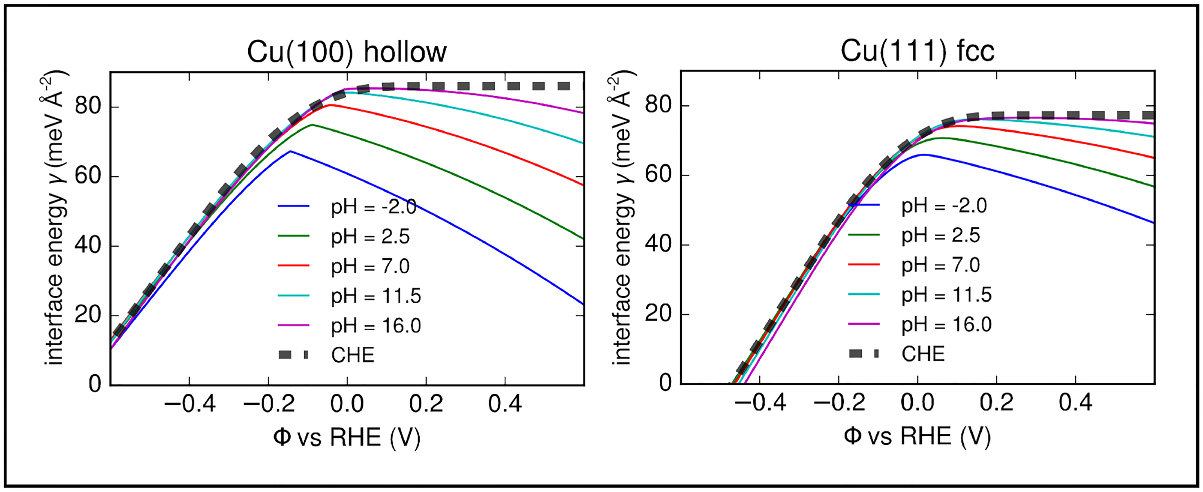

Supplementary Figure 18: pH dependence of interface energies on the RHE scale. Included is also the commonly used zero-net-charge evaluation according to the computational hydrogen electrode model (dashed grey line, marked as CHE).

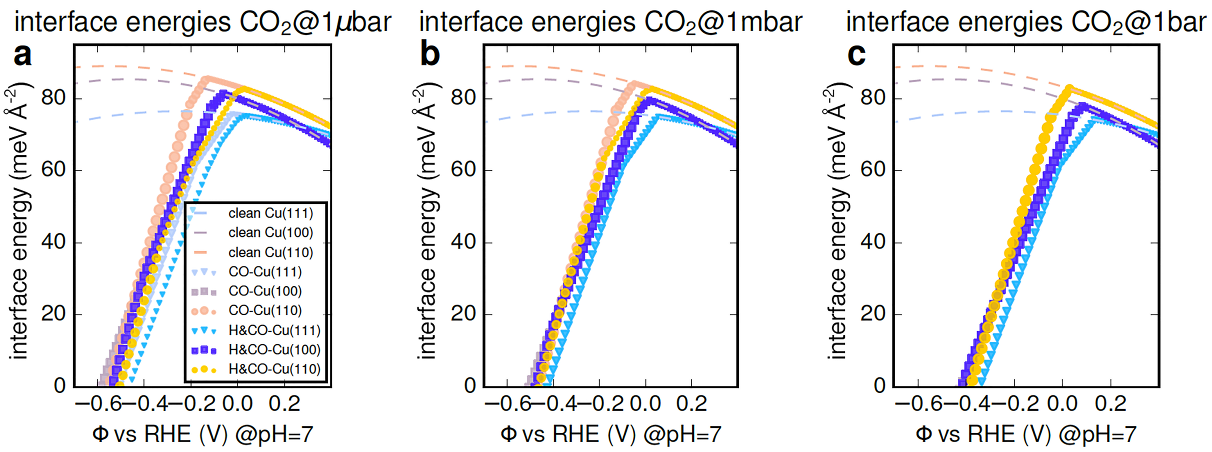

Supplementary Figure 19: Interface energies at pH 7. **a-c** Interface energies on the RHE scale for CO and H&CO covered surfaces for increasing CO_2 pressures. The size of the datapoints indicates the absolute CO coverage. Changes in interface energy between CO and H&CO systems indicate the occurence of mixed or H covered surfaces. At 1 bar (subfigure **a**), all surfaces are expected to be purely CO covered also when protons are available. For intermediate **b** and low pressures **c** H covered and coadsorbed terminations are observed (see also Supplementary Fig. 20). However the overall interface energy trends are very independent of the details of the surface termination.

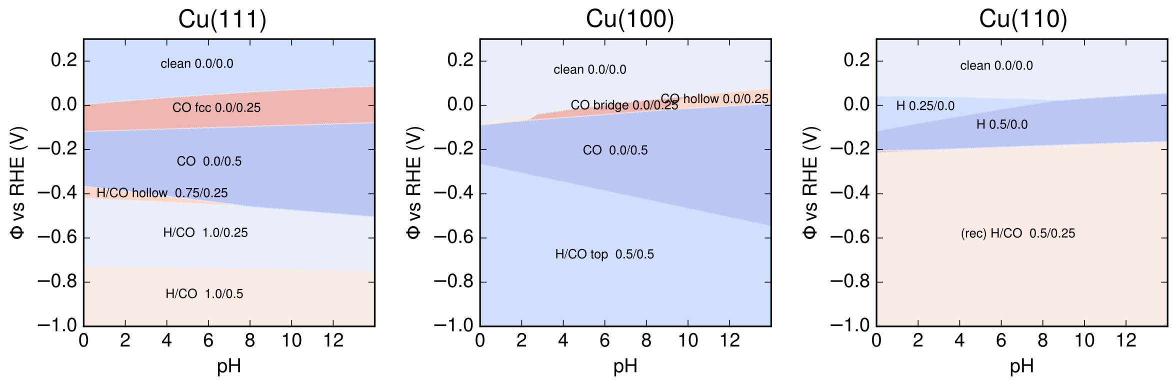

Supplementary Figure 20: Surface Pourbaix diagram on the RHE scale. The stable surface terminations are determined for CO deriving from CO_2 at a chemical potential corresponding to a pressure of 1 mbar. As indicated above, coadsorbed H&CO terminations occur for lower potentials in agreement with the experimentally observed simultaneous evolution of H and hydrocarbons. For potentials larger than -0.4 V Cu(111) and Cu(100) are predominantly covered with CO. The suppression of H evolution due to site blocking of CO has been reported experimentally [6].

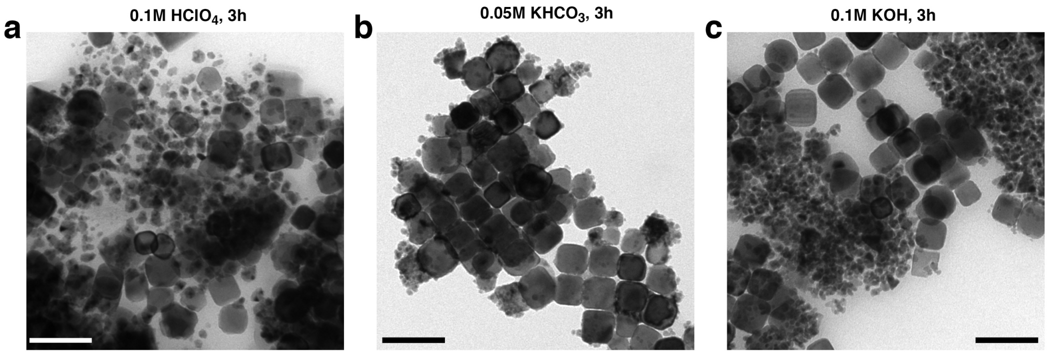

Supplementary Figure 21: Nanoclustering of the CuNCs in various electrolytes. CuNCs electrolyzed for 3 h in **a** 0.1 M $HClO_4$ (pH = 1), **b** 0.05M $KHCO_3$ (pH = 6.8 but lower buffer capacity), and **c** 0.1 M KOH (pH = 13), under the typical CO_2RR conditions. Scale bars: 100 nm.

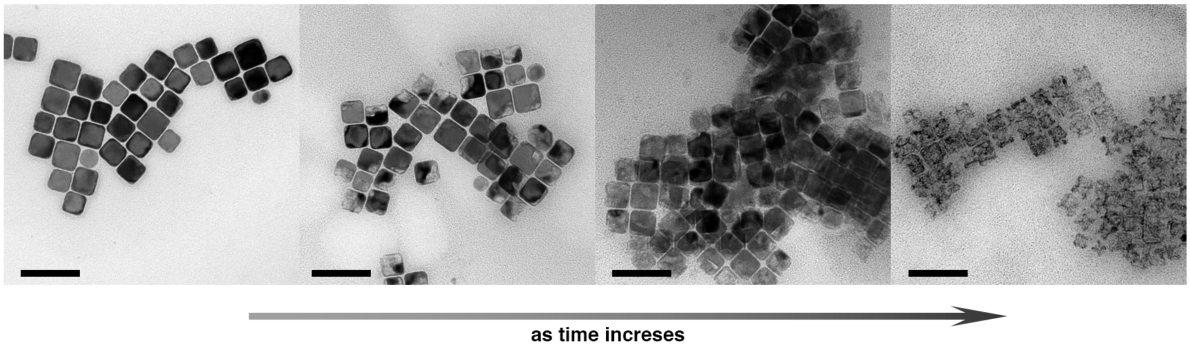

Supplementary Figure 22: Dissolution of CuNCs by a voltage. TEM images of CuNCs that were deposited on TEM Cu grids and directly applied with a voltage of -1.1 V under ambient conditions for varied operation times up to 12 h, showing that CuNCs were broken up into nanoclusters. Scale bars: 100 nm.

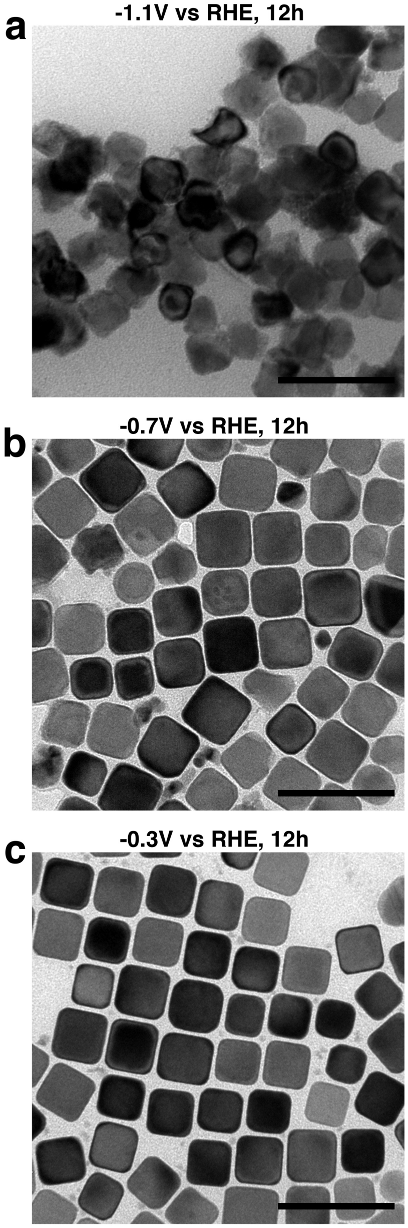

Supplementary Figure 23: Mitigation of nanoscale structural changes at less negative potential. **a-c** TEM images of 41 nm CuNCs that were collected after electrolysis under typical CO_2RR conditions for 12 h, at a potential of **a** -1.1 V, **b** -0.7 V, and **c** -0.3 V vs. RHE, showing less degradation of CuNCs at more positive potentials. The products at potentials of -0.7 V and -0.3 V are primarily CO, H_2 and formate. Scale bars: 100 nm.

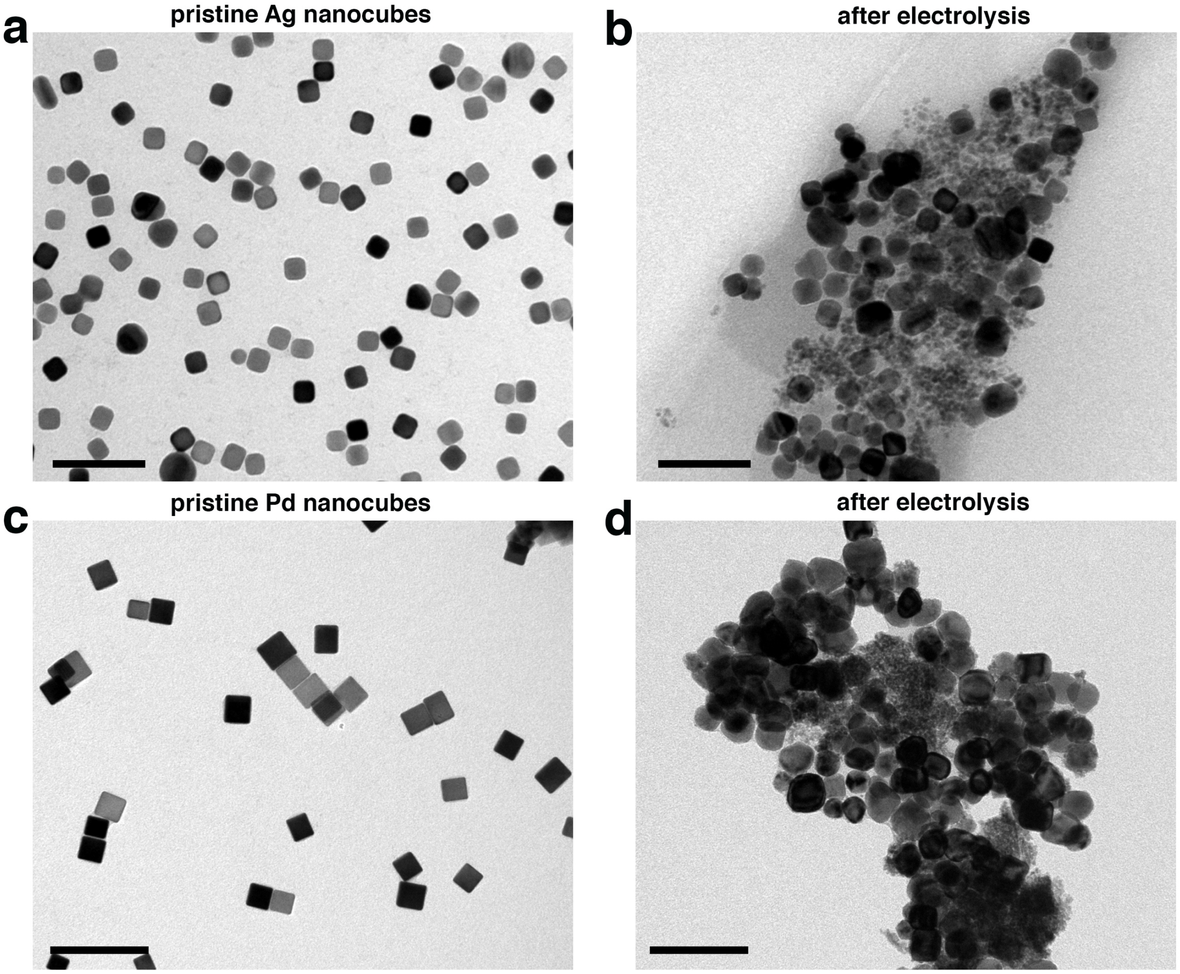

Supplementary Figure 24: Extension of nanoclustering to Ag nanocubes and Pd nanocubes. TEM images of **a, b** Ag nanocubes and **c, d** Pd nanocubes **a, c** before and **b, d** after electrolysis at -1.1 V and -1.7 V vs RHE, respectively, for 2 h, showing that nanoclustering also occurs in Ag- and Pd-based electrocatalysts. Here, the voltage applied for Pd nanocubes was a bit more negative than normal ones employed CO2RR. For the Pd nanocubes, nanoclustering was observed at -1.7 V vs RHE instead of -1.1 V. This behaviour depends on the system-dependent energetics. Nevertheless, it proves the generality of potential-induced nano clustering in metallic electrocatalysts. Scale bars: 100 nm.

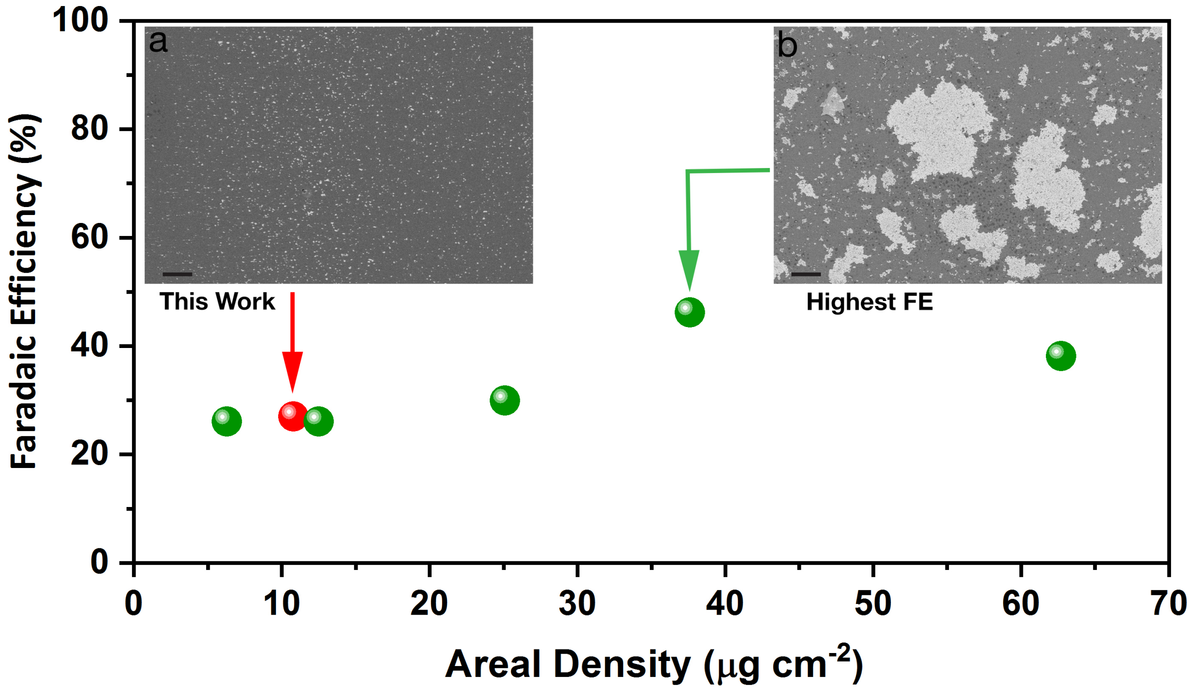

Supplementary Figure 25: Loading-dependent Faradaic efficiency. Ethylene Faradaic efficiency of 41 nm CuNCs as a function of areal density. Insets are SEM images of the samples with **a** the areal density used in this work and **b** yielding the highest FE. Scale bars: 20 μm.

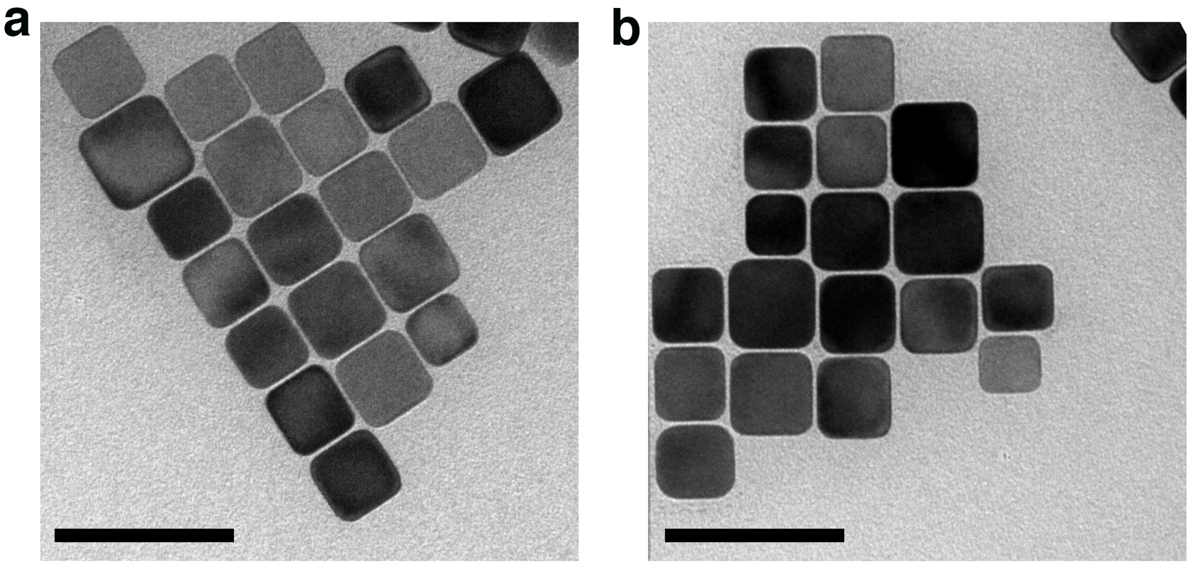

Supplementary Figure 26: Influence of transfer method on particle structure. **a, b** TEM images of CuNCs that were **a** directly deposited on a TEM grid and **b** first deposited on a glassy carbon substrate and then transferred to a TEM grid. The unchanged morphology demonstrates that such a transfer method does not affect the particle shape and splitting. Scale bars: 100 nm.

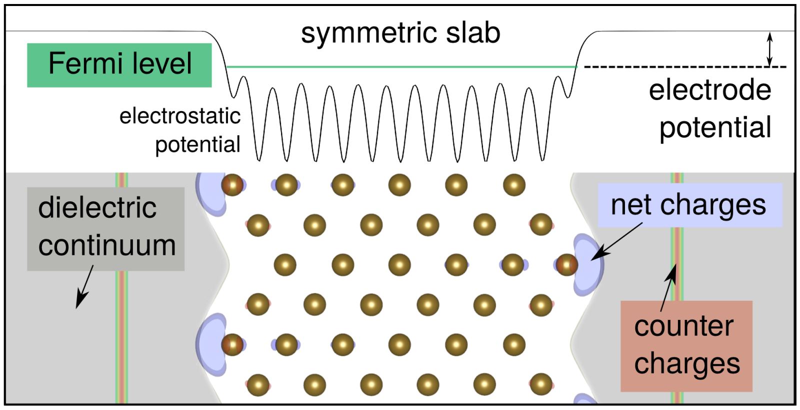

Supplementary Figure 27: Schematics of the computational setup. Symmetric slab calculations within the SCCS setup with a quantum mechanical and classical dielectric region. Net charges due to extra electrons $N(e)^{net}$ are compensated by an equal amount of planar counter charges in the dielectric region, such that the electrostatic potential flattens and the total energy converges.

Supplementary Tables

Supplementary Table 1: Properties of pristine Cu surfaces in implicit water. Reported are the theoretically determined potential of zero charge (PZC) on the absolute scale. The PZC corresponds to the workfunction in water, determined from a clean slab. PZC[=max(γ)] is the potential where the interfacial energy has a maximum (Lippmann's equation). In the SCCS setup this is slightly different than the PZC, due to the fact, that we interpolate intefacial energies with a second order polynomial although however the SCCS model exhibits potential dependent interfacial double layer capacitances, such that the exact PZC can't be reproduced. However, we reduce capacitance-drift-induced energy errors, which we think is more important, also because the inaccuracies in PZC are quite small. Interface energies at the PZC are reported in the column max(γ) and interfacial double layer capacitances in column capacitance@max(γ). Experimental PZC taken from references in Ref. [7].

surface	PZC	PZC[=max(γ)]	PZC[Exp.]	max(γ)	capacitance@max(γ)
units	V	V	V	meVÅ^{-2}	μF cm^{-2}
Cu(111)	3.88	3.77	4.24	77	50
Cu(100)	3.59	3.55	3.90	85	76
Cu(100)(5x1)	3.73	3.72	3.90	100	54
Cu(110)	3.45	3.43	3.76	88	42
Cu(110)(2x1)	3.51	3.47	3.76	89	58

Supplementary Table 2: Summary of the considered surfaces. Reported is the surface energy γ at the potential of zero charge (PZC), the PZC on the absolute scale (= workfunction of the non-charged slab in SCCS water) and the interfacial double layer capacitance at PZC.

surface<br>conditions<br>units	γ<br>@[PZC,pH=0]<br>meVÅ^{-2}	abs. PZC<br>-<br>V	capacitance<br>@[PZC]<br>μF cm^{-2}	sideview<br>-<br>-	topview<br>-<br>-
Cu(111)fcc/top;<br>Θ=2.0	53.0	3.633	25	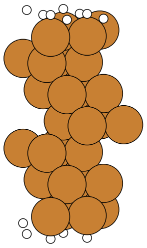	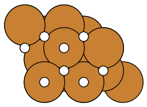
Cu(111)fcc/top;<br>Θ=1.25	27.2	3.889	26	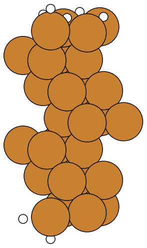	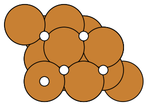
Cu(111)fcc/hcp;<br>Θ=2.0	-110.7	3.012	21	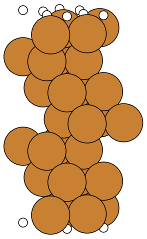	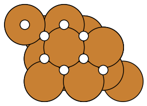
Cu(111)fcc/hcp;<br>Θ=1.25	-49.5	3.542	21	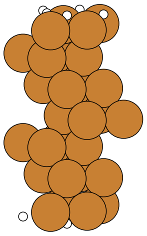	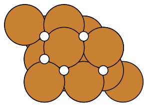

Cu(111)bridge;<br>$\Theta=0.25$	54.5	3.858	38	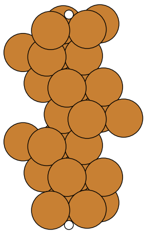	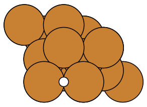
Cu(111)bridge;<br>$\Theta=0.75$	66.6	4.184	22	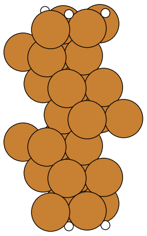	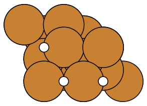
Cu(111)bridge;<br>$\Theta=0.5$	57.7	4.074	29	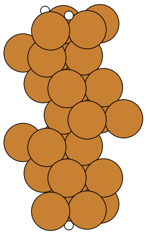	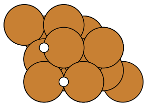
Cu(111)bridge;<br>$\Theta=1.0$	114.4	4.386	21	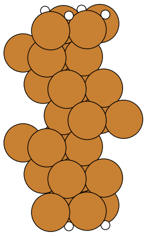	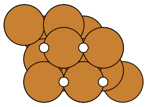
Cu(111)top;<br>$\Theta=0.25$	70.7	3.862	42	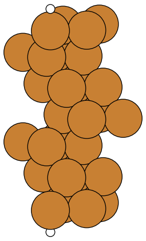	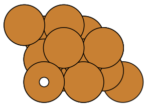

Cu(111)top; Θ=0.75	192.1	4.51	33	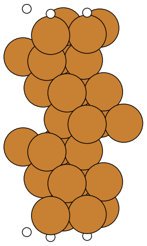	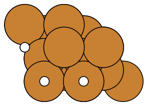
Cu(111)top; Θ=0.5	109.3	4.169	37	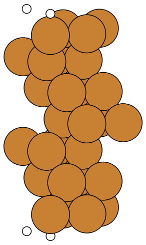	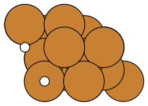
Cu(111)top; Θ=1.0	307.2	4.82	24	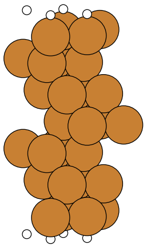	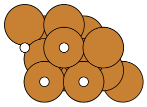
Cu(111)fcc; Θ=0.25	48.9	3.86	37	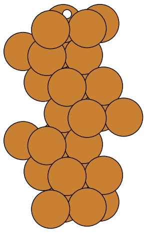	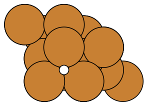
Cu(111)fcc; Θ=0.75	16.3	3.981	22	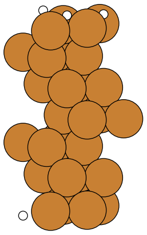	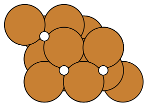
Cu(111)fcc; Θ=0.5	29.5	3.935	28	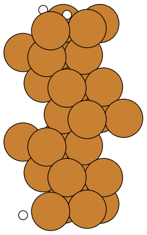	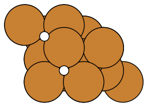

Cu(111)fcc; Θ=1.0	21.2	4.089	20	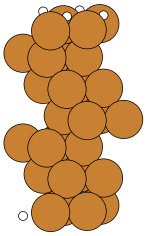	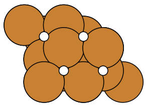
Cu(111)	76.3	3.884	86	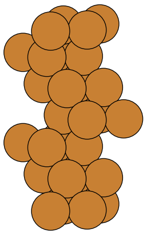	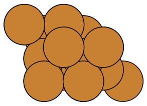
Cu(111)hcp; Θ=0.25	51.1	3.897	38	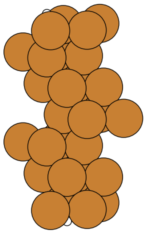	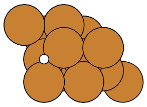
Cu(111)hcp; Θ=0.75	17.8	3.982	23	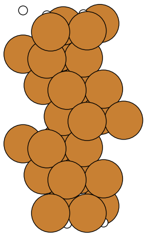	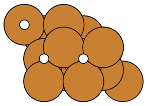
Cu(111)hcp; Θ=0.5	36.0	3.988	29	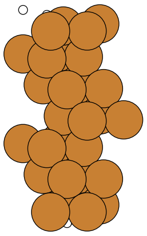	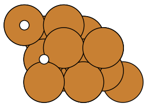
Cu(111)hcp; Θ=1.0	24.7	4.103	20	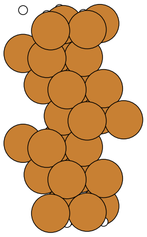	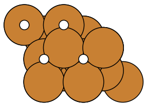

Cu(111)CO bridge 0.0/0.25	35.3	3.995	30.0	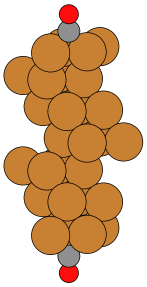	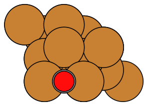
Cu(111)CO top 0.0/0.25	-1.0	3.543	30.0	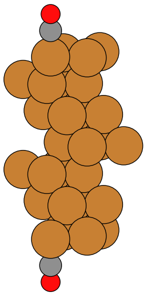	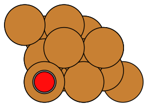
Cu(111)CO fcc 0.0/0.25	39.1	4.088	30.0	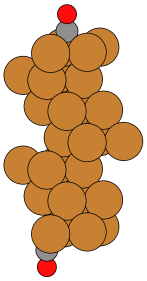	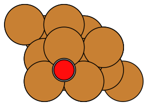
Cu(111)CO hollow 0.0/0.25	38.4	4.069	30.0	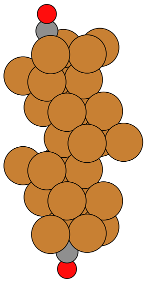	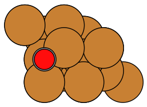

Cu(111)H/CO 1.0/0.25	-61.2	3.776	30.0	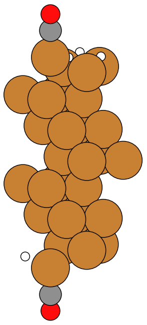	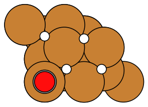
Cu(111)H/CO 1.0/0.5	-90.5	3.678	30.0	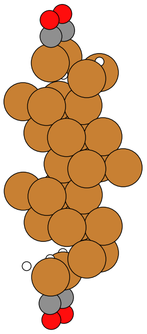	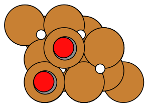
Cu(111)H/CO top 0.75/0.25	-67.1	3.681	30.0	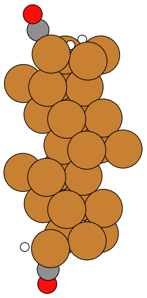	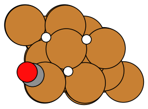
Cu(111)H/CO top 0.5/0.5	-17.7	3.851	30.0	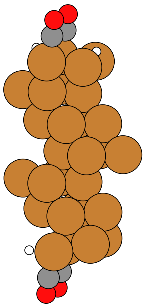	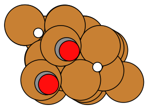

Cu(111)CO 0.0/0.5	91.1	4.388	30.0	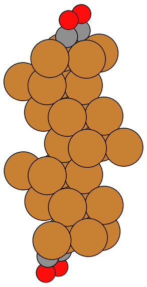	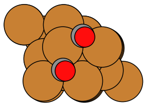
Cu(111)CO 0.0/0.5	60.1	4.327	30.0	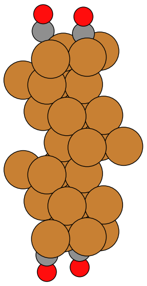	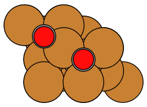
Cu(111)H/CO hol-low 0.75/0.25	-13.8	3.944	30.0	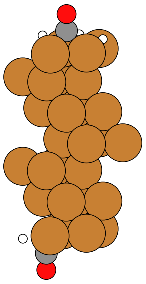	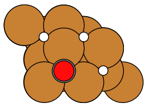
Cu(111)H/CO hol-low 0.5/0.5	94.0	4.29	30.0	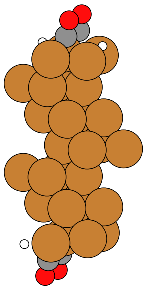	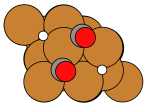

Cu(100)hollow; Θ=1.0	-49.0	3.436	25	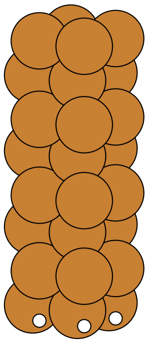	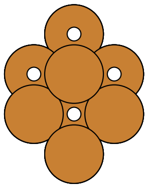
Cu(100)hollow/top; Θ=2.0	577.5	5.367	27	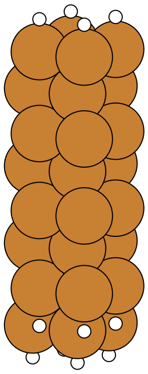	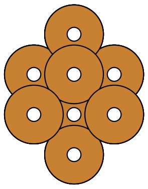
Cu(100)hollow/top; Θ=1.25	38.0	3.908	37	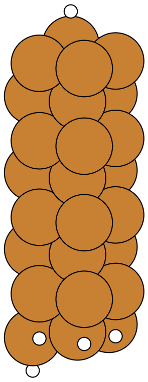	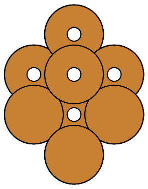

Cu(100)(5x1) rec.	99.6	3.731	51	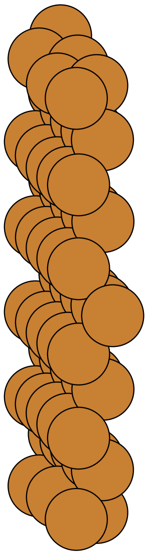	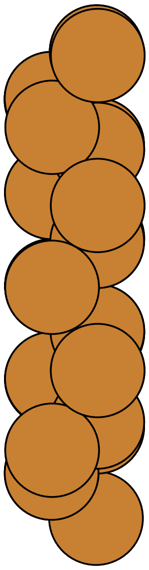
Cu(100)bridge; $\Theta=0.25$	59.9	3.683	45	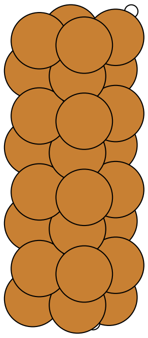	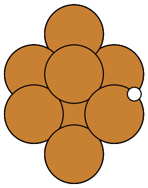
Cu(100)bridge; $\Theta=0.75$	86.8	4.27	31	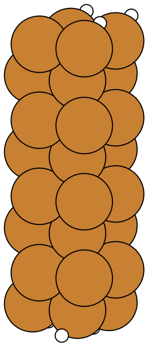	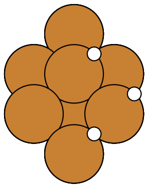

Cu(100)bridge; Θ=0.5	75.7	4.089	35	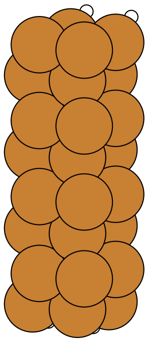	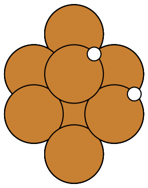
Cu(100)bridge; Θ=1.0	171.5	4.757	22	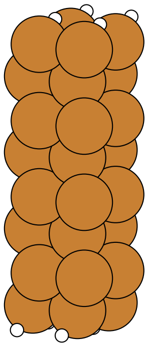	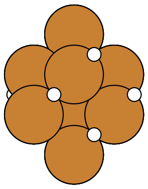
Cu(100)top; Θ=0.25	76.1	3.76	50	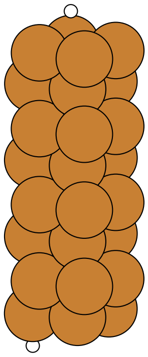	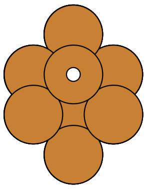
Cu(100)top; Θ=1.0	339.2	5.249	26	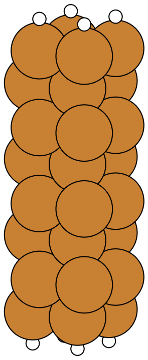	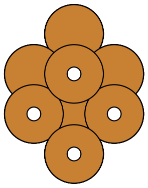

Cu(100)	85.4	3.586	65	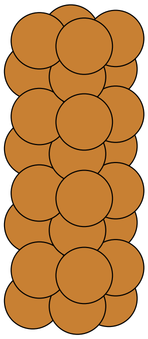	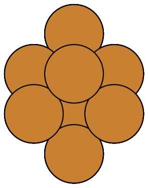
Cu(100)hollow; Θ=0.25	56.5	3.638	41	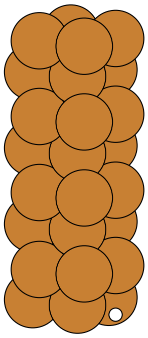	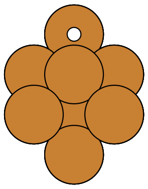
Cu(100)hollow; Θ=0.75	0.1	3.622	26	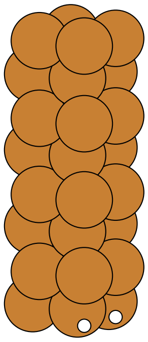	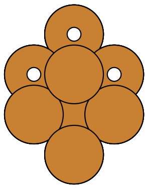
Cu(100)hollow; Θ=0.5	28.3	3.637	37	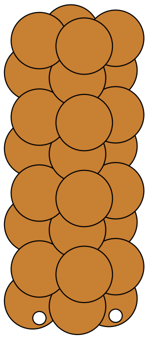	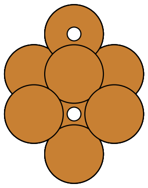

Cu(100)hollow; Θ=1.0	-49.0	3.436	25	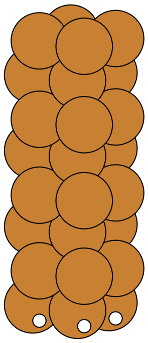	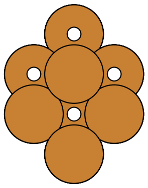
Cu(100)CO bridge 0.0/0.25	24.6	3.719	30.0	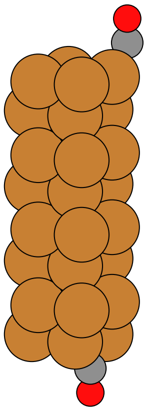	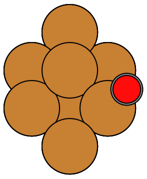
Cu(100)CO top 0.0/0.25	10.1	3.496	30.0	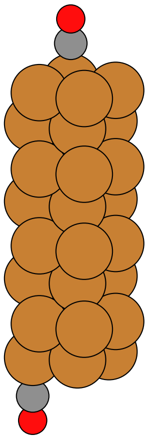	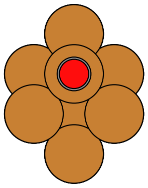

Cu(100)CO hollow 0.0/0.25	35.5	3.866	30.0	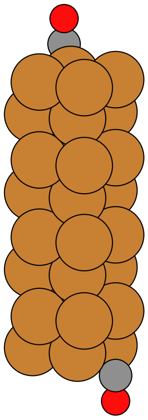	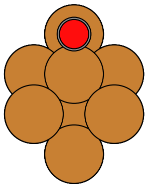
Cu(100)H/CO top 0.75/0.25	-108.0	3.302	30.0	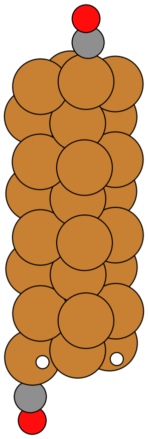	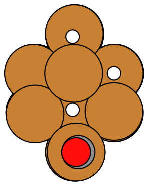
Cu(100)H/CO top 0.5/0.5	-162.7	3.251	30.0	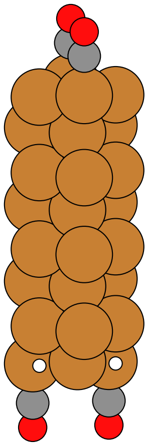	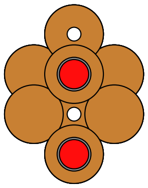

Cu(100)CO 0.0/0.5	58.9	4.286	30.0	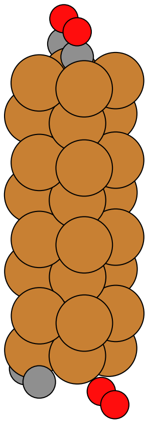	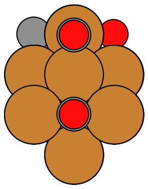
Cu(100)H/CO hol-low 0.75/0.25	-94.9	3.387	30.0	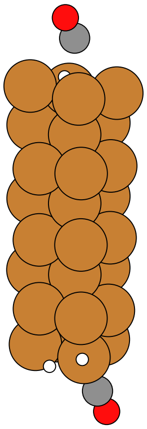	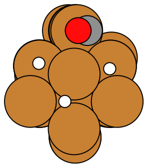
Cu(110) (2x1) rec.	89.0	3.505	70	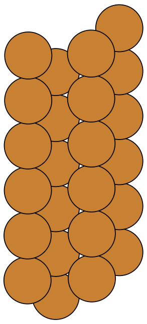	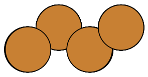
Cu(110)	88.1	3.446	98	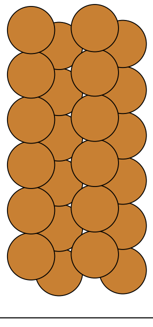	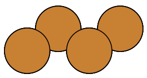

Cu(110)top ; Θ=0.25	32.5	3.375	49	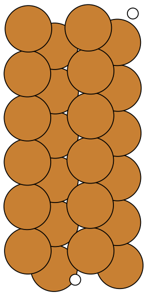	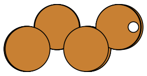
Cu(110)hollow ; Θ=0.25	37.0	3.328	47	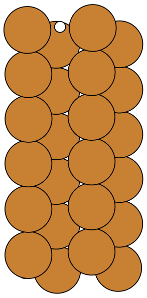	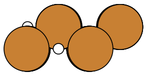
Cu(110)top ; Θ=0.75	99.2	4.173	29	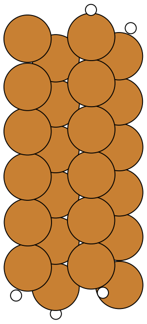	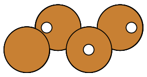
Cu(110)bridge ; Θ=0.75	48.8	4.113	24	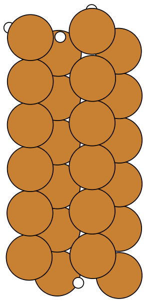	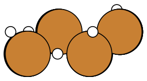

Cu(110)top ; Θ=0.5	-10.7	3.137	34	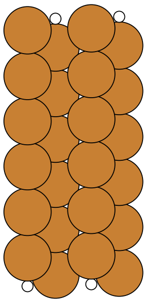	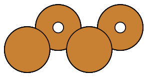
Cu(110)bridge ; Θ=0.5	48.3	3.974	31	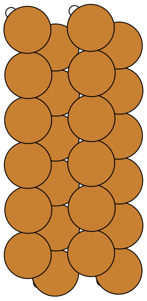	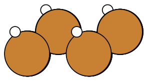
Cu(110)hollow ; Θ=0.5	-36.4	3.119	41	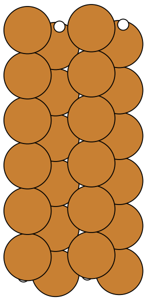	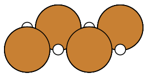
Cu(110)top ; Θ=1.0	374.9	5.077	28	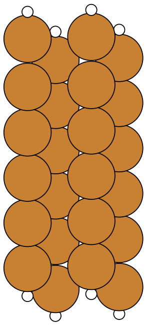	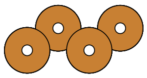

Cu(110)bridge ; Θ=1.0	208.9	4.831	24	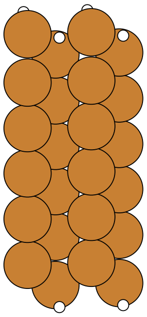	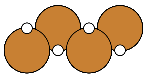
Cu(110)hollow ; Θ=1.0	-37.6	3.599	22	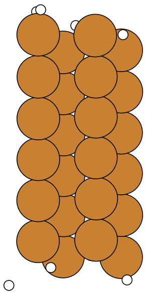	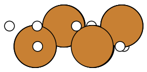
Cu(110)(unrec) 0.0/0.25	-9.6	3.497	30.0	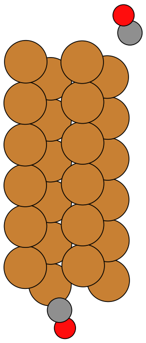	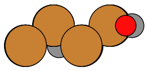
Cu(110)(unrec) 0.25/0.25	16.6	3.936	30.0	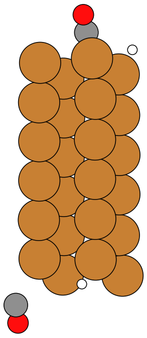	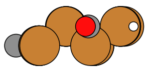

Cu(110)(unrec) 0.25/0.25	17.5	3.944	30.0	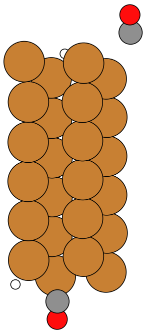	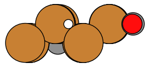
Cu(110)(unrec) 0.0/0.5	3.1	3.961	30.0	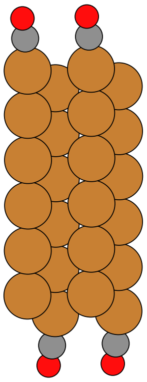	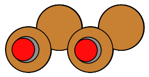
Cu(110)(unrec) 0.0/0.5	98.2	4.41	30.0	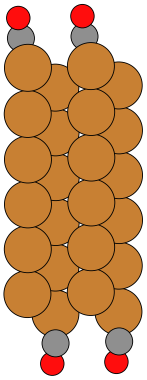	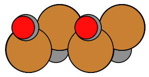
Cu(110)(unrec) 0.0/0.25	-11.1	3.447	30.0	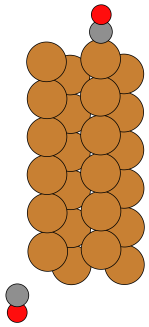	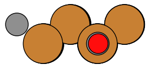

Cu(110)(unrec) 0.25/0.25	14.5	3.923	30.0	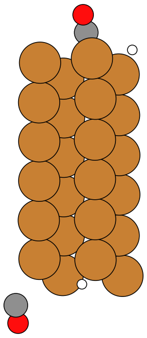	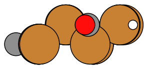
Cu(110)(unrec) 0.25/0.25	20.9	3.957	30.0	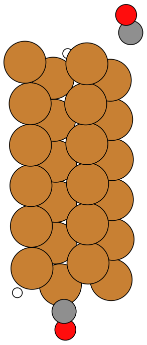	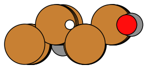
Cu(110)(unrec) 0.25/0.25	-9.7	3.763	30.0	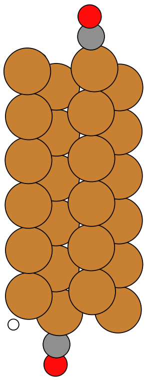	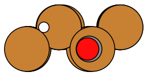
Cu(110)(unrec) 0.0/0.25	-12.1	3.439	30.0	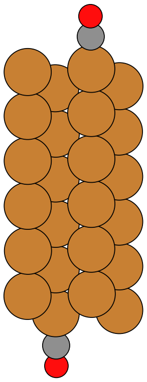	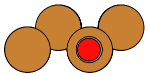

Cu(110)(unrec) 0.5/0.25	-81.0	3.534	30.0	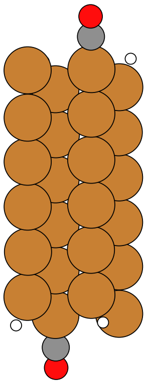	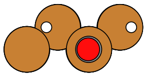
Cu(110)(unrec) 0.0/0.5	55.3	4.21	30.0	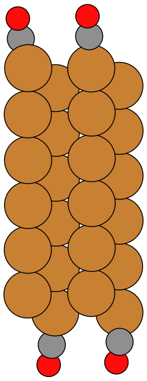	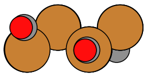
Cu(110)(unrec) 0.0/0.25	-8.4	3.514	30.0	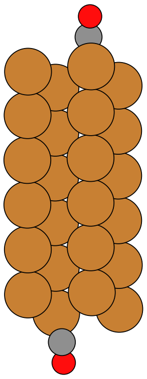	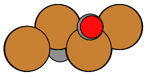

Cu(110)(unrec) 0.25/0.25	25.1	3.99	30.0	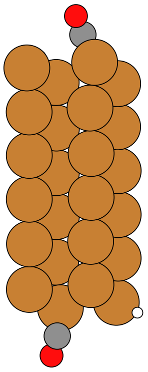	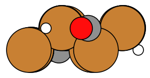
Cu(110)(2x1) rec. bridge ; Θ=0.25	47.2	3.696	45	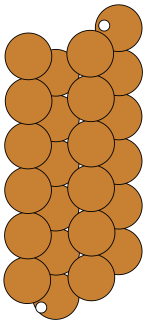	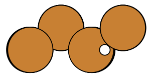
Cu(110) (2x1) rec. bridge ; Θ=0.75	-79.5	3.309	22	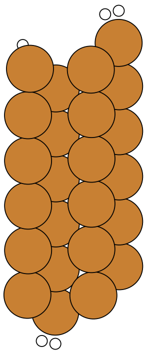	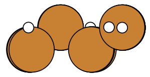
Cu(110) (2x1) rec. bridge ; Θ=0.75	2.4	3.88	27	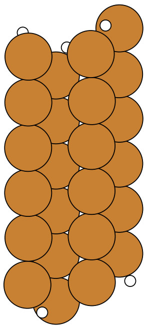	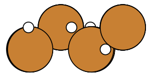

Cu(110) (2x1) rec. bridge ; Θ=0.5	18.8	3.849	29	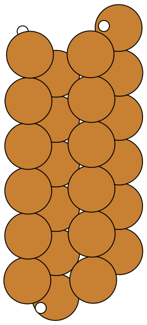	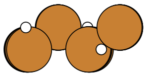
Cu(110) (2x1) rec. top ; Θ=0.5	21.8	3.803	28	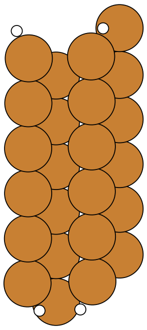	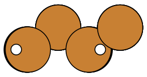
Cu(110)(rec) H/CO 0.25/0.25	33.3	4.055	30.0	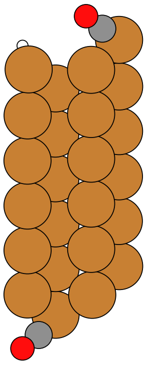	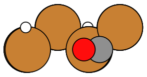
Cu(110)(rec) H/CO 0.25/0.25	32.7	4.053	30.0	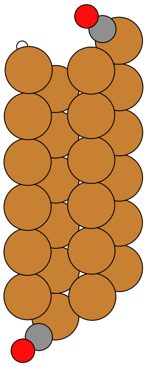	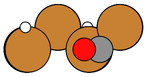

Cu(110)(rec)<br>H/CO 0.25/0.25	33.2	4.056	30.0	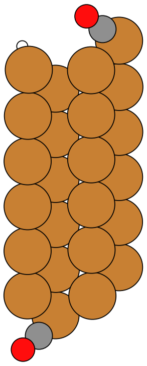	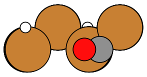
Cu(110)(rec)<br>H/CO 0.0/0.5	84.8	4.276	30.0	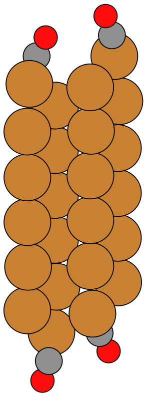	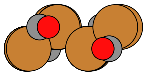
Cu(110)(rec)<br>H/CO 0.0/0.25	58.3	4.091	30.0	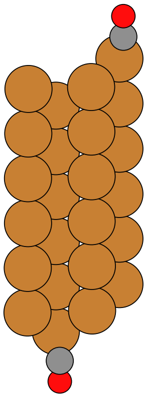	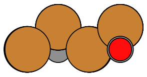

Cu(110)(rec)<br>H/CO 0.5/0.25	41.3	4.149	30.0	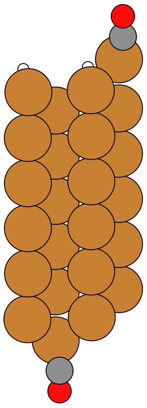	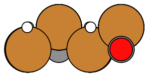
Cu(110)(rec)<br>H/CO 0.5/0.25	30.0	4.014	30.0	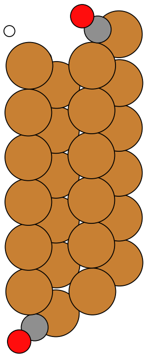	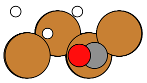
Cu(110)(rec)<br>H/CO 0.25/0.25	47.3	4.089	30.0	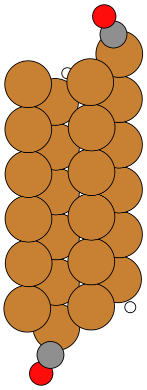	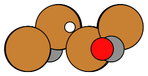

Cu(110)(rec) H/CO 0.0/0.25	64.9	4.117	30.0	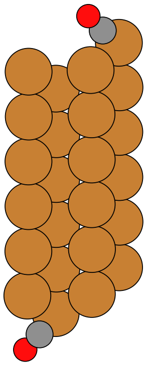	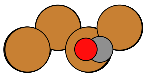
Cu(110)(rec) H/CO 0.5/0.25	44.7	4.167	30.0	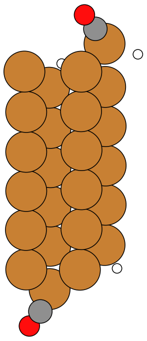	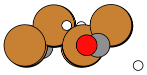

Supplementary Methods

EXPERIMENT

Chemicals

Oleylamine (70%), trioctylphosphine (TOP, 90%), trioctylphosphine oxide (TOPO, 90%), copper (I) chloride (CuCl, 99.99%), copper (I) bromide (CuBr, 99.999%), diethylene glycol (DEG, $\geq$99.0%), sodium hydrosulfide hydrate ($NaHS{\cdot}xH_2O$), aqueous hydrochloric acid solution (HCl, 37%), poly (vinyl pyrrolidone) (PVP, $M_W \approx 55\ 000$), silver trifluoroacetate (CF_3COOAg, $\geq$99.99%), hexadecyltrimethylammonium bromide (CTAB, 99%), ascorbic acid (99%), potassium carbonate (K_2CO_3, 99%), ethanol (95+%, anhydrous), hexane (95%, anhydrous) were all purchased from Sigma-Aldrich and used as received without further purification. All aqueous solutions were prepared using deionized (DI) water with a resistivity of 18.2 MΩ·cm. A H_2PdCl_4 solution (10 mM) was prepared by dissolving 0.1773 g of palladium(II) chloride ($PdCl_2$, $>$98.0%, TCI) in 10 mL of 0.2 M HCl solution and then diluting to 100 mL with DI water.

Synthesis of Ag nanocubes, Pd nanocubes, Cu octahedrons and Cu nanospheres

Ag nanocubes were synthesized according to reported procedures [8]. Briefly, 6.5 mL of DEG was first added into a flask and heated to 140 °C for 30 min under magnetic stirring. Reagents that were separately dissolved in DEG, i.e. NaSH (0.06 mL, 3 mM), HCl (1.025 mL, 3 mM), and PVP (1.25 mL, 20 mg/mL), were then sequentially injected and allowed to be heated for 4, 2, and 2 min, respectively. Finally, CF_3COOAg (0.4 mL, 282 mM) was introduced and the resultant mixture was heated for another 1.5 h before it was quenched with an ice-water bath. The products were collected by centrifugation, followed by washing with acetone and then deionized water.

Pd nanocubes were synthesized following an aqueous-based method [9]. Specifically, a H_2PdCl_4 solution (1 mL, 10 mM) was first mixed with a CTAB solution (20 mL, 12.5 mM) under stirring. The mixture was then heated at 95 °C for 5 min, followed with an addition of ascorbic acid solution (0.16 mL, 100 mM) and heating for another 20 min. The obtained cubes were washed by three times of centrifugation in water.

Cu octahedrons were synthesized following reported protocols [10]. Specifically, a precursor solution

of CuCl (0.2 mmol) and TOP (2 mL) was preheated at 200 °C for 2 h. In the meantime, 18 mL of oleylamine was purged under strong magnetic stirring with a N_2 flow for 40 min. Then, the oleylamine was heated to 335 °C followed by a quick injection of the hot precursor solution. After 20 min, the resulting colloidal products were cooled to room temperature rapidly with a water-bath and washed by centrifugation.

Cu nanospheres ($\approx$ 7.4 nm) were synthesized following exactly the same procedure as that reported in our previous paper [11].

Electrocatalytic Measurements

Platinum foil and Ag/AgCl electrode (leak free series, Innovative Instruments, Inc.) were used as the counter electrode and reference electrode, respectively. Glassy carbon plates (2.5 cm x 2.5 cm, Type 2, Alfa Aesar) loaded with the electrocatalysts (i.e., CuNCs) served as the working electrode. Before loading electrocatalysts, glassy carbons were typically polished using 1 μm diamond and 50 nm alumina (BAS), followed by a rinse with Milli-Q water, ultrasonication in acetone for 5 min, and blowing dry with N_2. A Selemion anion exchange membrane was used to separate the anodic and cathodic compartments. Each compartment was filled with a CO_2-satureated $KHCO_3$ solution (2 mL, 0.1 M) as an electrolyte. Such a $KHCO_3$ solution was prepared by bubbling a freshly prepared K_2CO_3 solution (0.05 M) with CO_2 for 30 min.

For gas product analysis, a gas chromatograph (GC, SRI instruments) equipped with a HayeSep D porous polymer column, thermal conductivity detector, and flame ionization detector was used. Ultra-high purity N_2 (99.999%) was used as a carrier gas. The concentration of gaseous products was determined using calibration curves from standard gases. For liquid product analysis, a high-performance liquid chromatography (HPLC) on an UltiMate 3000 instrument from Thermo Scientific was used.

The calculation of Faradaic efficiency (FE) for gas products was performed using following equation:

$$FE = \frac{n \cdot F \cdot ppm \cdot G \cdot P}{R \cdot T \cdot I} \quad (1)$$

Where, n is the number of electrons transferred to product formation from CO_2 (e.g., 2 for H_2 and 12 for C_2H_4), F the Faraday constant (96485 C mol^{-1}), ppm the measured concentration of the product by GC, G gas flow rate, I the cell current, P = 1.01 X 10^5 Pa, R = 8.314 J mol^{-1} K^{-1}, T = 273.15 K.

The faradaic efficiencies of liquid products were calculated as follows:

$$FE = \frac{n \cdot M \cdot V \cdot F}{Q} \tag{2}$$

Where, n is the number of electrons transferred to CO_2 to produce a given product, M the Molar concentration of the liquid product, V the liquid volume, F the Faraday constant, and Q the total charge passed over a given time of analysis.

THEORETICAL DFT CALCULATIONS

Grand canonical DFT calculations

An electrochemical interface is defined as an interface between an electron and an ion conductor. We model the interface by an implicit solvation model, where the metal substrate (Cu) and potential adsorbates (H, CO) are treated quantum mechanically (QM) using a slab geometry and the solution is represented by a polarizable continuum (PC). The QM region is embedded as a cavity of dielectric constant of 1 inside a PC of the dielectric constant of water (78.3) (see Supplementary Fig. 27). The geometry of the cavity is determined self-consistently based on the electronic charge density using a smooth switching function appropriate for plane wave DFT codes (SCCS, [12]). The calculations are performed using the ENVIRON module [13, 14], which implements the SCCS model in the open source code Quantum ESPRESSO [15]. In such a setup, the electrostatic potential within the dielectric continuum can serve as an absolute reference for the electrostatic potential [7]. The electrode potential can be changed by increasing or reducing the total number of electrons in the system and measuring the shift of the Fermi level with respect to the flat Hartree potential in the solution (Supplementary Fig. 27). In reality and in the simulation these net charges are compensated by counter charges (ions) in the electrochemical double layer inside the solution. Here, we use simple 2D planar counter charges with a gaussian width of 0.5 a.u. (a functionality of ENVIRON) (Supplementary Fig. 27), which are introduced on both sides of the slab at a distance of 5 Å. The necessary charge compensation can also be achieved e.g. by explicit counter ions in explicit water [16] or using a Poisson-Boltzmann description (or related approaches) for the ions in solution [13, 7, 17, 18]. Both approaches are, however, computationally more expensive and can also exhibit arbitrariness e.g. in ion concentrations or exclusion volumes. Furthermore, we do not introduce any explicit water molecules as it is still unclear, in our opinion, how to consistently separate the dielectric screening at the interface into partly due to interfacial water and partly due to bulk water. Furthermore, in such an explicit-implicit water setup the electrode potential becomes strongly dependent on the orientation of interfacial water molecules and, in particular, would again necessitate thermodynamic sampling e.g. by molecular dynamics which we wanted to avoid.

The onset and width of the dielectric continuum depends on two parameters ρ_{min} and ρ_{max} , which we determined in a separate study (to be published), by enforcing to reproduce the theoretically determined potential of zero charge (PZC) of Pt(111) of approx. 4.98 V [19]. The values are $\rho_{\mathrm{min}} = 0.0013$ and $\rho_{\mathrm{max}} = 0.01025$ and are very close to the average of the parameters, appropriate for correct solvation energies of cations and anions [20]. Detailed results on this will be published elsewhere.

In this study, we use only purely electrostatic coupling and neglect energy contributions due to

cavity formation as these cancel largely for 2D setups / slabs. We compared PZC and interfacial capacitances to experimental results for (111), (100), (5x1) reconstructured (100), (110) and (2x1) reconstructured (110) surfaces of Pt, Au, Ag and Cu and find good agreement with experimental values. For Cu the inaccuracies are slightly larger as for the other coinage metals. The interfacial capacitances are too large (42-76 μF cm^{-2} vs $\approx$ 30 μF cm^{-2}) for the pristine surfaces, they reduce, however, to experimental values for the H covered surfaces (see Supplementary Table 1 and 2). PZC of the pristine surfaces are underestimated by $\approx$ 0.3 V (see Supplementary Table 1). On the other hand, also vacuum workfunctions as determined with standard DFT exchange-correlation functionals can exhibit errors of this size. In summary, the inaccuracy of our potential scale is of the order of 0.3 V.

The thermodynamic properties of an electrochemical interface are determined by the electrochemical potentials of the involved species. In our case, these are the slab Cu atoms $N(\mathrm{Cu})$, the adsorbed protons $N(\mathrm{H}^+)$ and CO molecules $N(\mathrm{CO})$ and the electrons $N(\mathrm{e})$, which can be exchanged with the external circuit. It is convenient to introduce, in addition, the terms *net surface charges* $N(\mathrm{e})^{\mathrm{net}}$ (surface excess charges) which are the charges beyond charge neutrality, and the number of electrons $N(\mathrm{e})^{\mathrm{abs}}$ exchanged with the external circuit upon ion adsorption. For proton adsorption, $N(\mathrm{e})^{\mathrm{abs}}$ can be calculated from the net surface charges $N(\mathrm{e})^{\mathrm{net}}$ and the adsorbed protons $N(\mathrm{H}^+)$ via:

$$N(\mathrm{e})^{\mathrm{abs}} = N(\mathrm{e})^{\mathrm{net}} + N(\mathrm{H}^+) \tag{3}$$

In the following we focus on the description of the treatment of proton adsorption. The treatment of CO is a straight forward extension with minor differences and is discussed briefly subsequently.

Analogously to ab-initio thermodynamics of solid-gas interfaces [21], the formation energy in electrochemical variable charge calculations in slab geometry with the configuration ($N(\mathrm{Cu})$, $N(\mathrm{H}^+)$, $N(\mathrm{e})^{\mathrm{abs}}$) is evaluated by a fictitious coupling to a thermodynamic bath, which supplies the involved components at the (electro-)chemical potentials ($\mu(\mathrm{Cu})$, $\tilde{\mu}(\mathrm{H}^+)$, $\tilde{\mu}(\mathrm{e}^-)$). As indicated by the tilde, the electrons and the adsorbate - the protons - are charged species inside the reservoirs (the external circuit and the solution), such that the electrochemical potential has to be used. The numerical value of the individual electrochemical potentials depends on the chosen reference of the electrostatic potential. As indicated before, we chose here the flat region of the Hartree potential inside the dielectric continuum as a reference, such that the electron electrochemical potential $\tilde{\mu}(\mathrm{e}^-)$ is equal to the workfunction in this system. In particular, it corresponds to the electrode potential Φ on the absolute scale as defined by Trasatti [22], because of the negligible water surface dipole potential, that is not included in implicit solvation models, and our chosen SCCS parametrization, which reproduces the PZC on the absolute scale. The electrochemical potential of a proton $\tilde{\mu}(\mathrm{H}^+)$ can be determined by the equilibrium conditions of the standard hydrogen electrode (SHE) at pH=0, for which we take the

experimental value 4.44 V. Assuming that Nernst law holds, it is given by:

$$\tilde{\mu}(\mathrm{H}^+) = [0.5\mu(H_2|\mathrm{g}) - k_B T \ln(10) \cdot \mathrm{pH} + 4.44\mathrm{eV}] \tag{4}$$

In principle, $\mu(H_2|\mathrm{g})$ is calculated from the classical partition function of an ideal molecular gas at the conditions (p = 1 bar, T = 298K) including rotational, translational and vibrational degrees of freedom based on the 0K DFT total energy, structure and vibrational modes. $\mu(\mathrm{Cu})$ is approximated by the 0K DFT energy per atom and assumed to be fixed as the bulk part of the Cu nanoparticles serves as the thermodynamic reservoir.

For the slab systems, we added to the DFT 0K energy a configurational entropy term $T\Delta S = N_s k_B T[\theta \ln\theta + (1-\theta)\ln(1-\theta)]$ of an ideal solution of adsorbates, where N_s is the number of accessible sites (number of surface atoms per cell) and $\theta = [N(\mathrm{H}^+)/N_s] \mod 1$ the coverage. Furthermore we approximate the vibrational contributions, by a readjustment using the H_2 vibrational properties, which means that we effectively neglect vibrational contributions for H_2 in equation 4.

Analogously to the ab-initio thermodynamics approach of surface science [21], the formation energy $\tilde{G}$ of a certain configuration ($N(\mathrm{Cu})$, $N(\mathrm{H}^+)$, $N(\mathrm{e})^{\mathrm{abs}}$) is evaluated via:

$$\begin{aligned}&\tilde{G}(\mu(\mathrm{Cu}), \tilde{\mu}(\mathrm{H}^+), \Phi | N(\mathrm{Cu}), N(\mathrm{H}^+), \mathrm{N(e)^{abs}}) = \mathrm{E_{DFT}}(\mathrm{N(Cu)}, \mathrm{N(H^+)}, \mathrm{N(e)^{abs}}) + \\ &T\Delta S - N(\mathrm{Cu})\mu(\mathrm{Cu}) - N(\mathrm{H}^+)\tilde{\mu}(\mathrm{H}^+) + N(\mathrm{e})^{\mathrm{abs}} e\Phi\end{aligned} \tag{5}$$

It should be noted that this operation corresponds effectively to a Legendre transform. Subsequent minimization with respect to the extensive variables ($N(\mathrm{H}^+)$, $N(\mathrm{e})^{\mathrm{abs}}$) results in the equilibrium compositions and the equilibrium grand potential G at given electrochemical potentials ($\tilde{\mu}(\mathrm{H}^+), \Phi$).

$$G(\tilde{\mu}(\mathrm{H}^+), \Phi) = \min_{N(H), N(\mathrm{e})^{\mathrm{abs}}} \left[\tilde{G}(\mu(\mathrm{Cu}), \tilde{\mu}(\mathrm{H}^+), \Phi)\right] \tag{6}$$

Interface energies are determined by normalization to the surface area $\gamma(\tilde{\mu}(\mathrm{H}^+), \Phi) = G(\tilde{\mu}(\mathrm{H}^+), \Phi)/2A$, considering that all calculations are performed in symmetric slab setups. A partial minimization with respect to $N(\mathrm{e})$ corresponds to interface energies at the respective potential Φ, for a fixed configuration of adsorbates.

An alternative, equally valid way to determine the equilibrium grand potential G, is to interpolate for each surface the dependence of the electrode potential on the number of electrons $N(\mathrm{e})^{\mathrm{abs}}$ and choose the configuration which realizes the target potential Φ, or, in other words, where the system's workfunction corresponds to Φ. The system's workfunction is given as the difference between the

Hartree potential far in the solution and the Fermi level, or alternatively, as the energy change upon infinitesimal electron exchange $\frac{\partial E_{\mathrm{DFT}}}{\partial N(\mathrm{e})}$ (Janak's theorem [23]). These two approaches are equivalent because:

$$\min_{N(\mathrm{e})^{\mathrm{abs}}} \left[\tilde{G}\right] \quad \leftrightarrow \quad \frac{\partial \tilde{G}}{\partial N(\mathrm{e})^{\mathrm{abs}}} = 0 \leftrightarrow \frac{\partial E_{\mathrm{DFT}}}{\partial N(\mathrm{e})} = -e\Phi = \epsilon_F \tag{7}$$

We have tested that both methods give equivalent results. The calculations and results are described below, more details on the methodology will be published in a separate paper.

The minimization of $\tilde{G}(\mu(\mathrm{Cu}), \tilde{\mu}(\mathrm{H}^+), \Phi|N(\mathrm{Cu}), N(H), N(\mathrm{e})^{\mathrm{net}})$ is performed numerically based on interpolated interfacial free energy landscapes $\tilde{G}$ which are constructed for a large collection of DFT calculations at fixed charge and interface composition using following protocol:

1. Perform DFT calculations in symmetric slab geometry for different surface charges and surface compositions. Different surface compositions correspond to different number of adsorbed H atoms, different adsorption sites or different H arrangements. Computational details and results are described below.
2. For each fixed interface composition, interpolate the potential dependence of the interface energy on Φ by a low order polynomial.
3. Interpolate the coverage dependence of the interface energy for individual adsorption sites on a fine potential grid by a low order polynomial. All analytic terms related to the pH are not interpolated but added subsequently in the evaluation.
4. Minimize the interpolated free energy landscapes with respect to coverage and adsorption site at the target pH, to obtain the equilibrium interface configuration (adsorption site, coverage and surface charge) and interface free energies.

The equilibrium nanoparticle shapes are evalutad by constructing the Wulff shapes using pymatgen [24] and their cubicity is evaluated by evaluating the relative amount of (100) surfaces.

For CO and mixed CO/H surface terminations we apply a methodology slightly different. We assume CO derives from CO_2 which has reacted with 2 protons to form a water molecule:

$$CO_2(g) + 2H^+ + 2e^- \rightarrow CO + H_2O(l) \tag{8}$$

As a result the chemical potential of CO $\tilde{\mu}(\mathrm{CO})$ can be expressed as a function of the (electro-)chemical potentials of CO_2, H^+ and e^-. $\mu(\mathrm{CO}_2)$ is calculated from the classical ideal gas partition function and corrected such as to reproduce the experimental CO oxidation energy. $\mu(\mathrm{CO}_2)$ depends on the CO_2 pressure, where it is not totally clear in how far chemical equilibrium is achieved

between dissolved and gaseous CO_2. As a result we performed calculations at several CO_2 pressures.

The involved 2 electrons involved in CO_2 reduction can be added to the absolute charge $N(\mathrm{e})^{\mathrm{abs}}$ (eq. 3) as well as the 2 protons to the proton term. Furthermore we simplified the determination of the potential dependence of interface energies for CO(/H) coverages. In fact, the potential dependence of the DFT energy can be estimated analytically by adding energy contributions due to capacitive charging of the electrochemical double layer. We tested this estimate for CO, H and Cl also on different substrates and find very good agreement. As a result, it is possible to extrapolate the Grand Potential interface energy from zero net charge calculations. A paper on this methodology is in preparation. Hence all results including CO molecules are obtained by this extrapolation method, using an interfacial capacitance of 30 μF cm^{-2}.

Computational details and results

General DFT parameters and bulk calculations

DFT calculations were performed using the plane wave code Quantum ESPRESSO [15] and the PBE flavour of the generalized gradient approximation to the exchange correlation functional.We use the suggested pseudopotentials from the excessively tested SSSP (effective) pseudopotential database [25], using a wavefunction cutoff of 45 Ry and charge density cutoff of 360 Ry.
Bulk properties of fcc Cu are determined from a variable cell relaxation using a Monkhorst-Pack k-point grid based on a k-point distance of 0.125 Å^{-1}. Brillouin zone integrations are performed using Marzari-Vanderbilt cold smearing [26] with a smearing width of 0.01 Ry. These parameters were chosen by analysing the convergence of the relative stability of Cu, Cu_2O_2, Cu_2O, Cu_2O_3, as well as of the H_2, O_2, H_2O molecules necessary for a consistent description of e.g. the Pourbaix diagram. Relative energies were converged up to 1 meV per atom when compared to highly accurate calculations at 65 Ry and a k-point grid with a spacing of 0.1 Å^{-1}. Our relaxed lattice constant is 3.6134 Å.

Pourbaix diagram calculations

The Pourbaix diagram of Cu is constructed using the functionality of pymatgen [24] and ASE [27]. For the stability of ions in water, we used the experimental formation energies as stored in ASE. The stability of copper containing phases in water is analysed by calculating total energies of all Cu, O, and H containing compounds as available from the Materials Project database [5] with a

stability of at least 0.1 eV above hull. Free energies of H_2O(g), H_2(g) and O_2(g) are determined from calculating the classical partition function of ideal molecular gases [21] including rotational, translational and vibrational degrees of freedom based on the 0K DFT total energy, structure and vibrational modes. As is common, the O_2(g) chemical potential (formation energy) is corrected by an appropriate amount, so as to reproduce the formation energy of liquid water under standard hydrogen electrode conditions (2.46 eV) and the equilibrium water vapor pressure of 0.035 bar [28]. An alternative approach was tested, as e.g. used in Materials Project, where both the oxygen and hydrogen chemical potentials are shifted [29] in order to reproduce both formation energies of water and of simple metal oxides (e.g. Na_2O, MgO, CaO, Al_2O_3) very accurately. However, inferior agreement with the experiment (Pourbaix Atlas) is found for this approach, also for other 3d metals. This is an important finding as it suggests more tests should be performed when applying energy corrections in phase diagram calculations.

The calculated Pourbaix diagram is displayed in Supplementary Fig. 15.

We found good agreement with the experimental Pourbaix diagram and the stability of metallic Cu in a potential window of approx. [-0.32 to 0.58 V vs RHE (pH 7)]. Whereas Cu gets oxidized for more positive potentials the CuH phase becomes stable at potentials below -0.3 V, which indicates the presence of H covered surfaces and hydride surface phases. CuH is in many publication not included as it is only metastable (higher pressures). In our opinion, however, the electrode potential represents an extremely large driving force for any reduction reaction e.g. CuH formation such that the normal phase stability considerations close to ambient conditions are not applicable. We wish to note, that we are not aware of any publication related to electrocatalytically active Cu, which treats specifically bulk CuH and (partially) H covered Cu surfaces as performed here, although significant amounts of H_2(g) are produced under reactive conditions, which is why we think these results can motivate new studies and improve our understanding of Cu catalysts in aqueous environments. A very recent publication on CuH nanoparticles supports indeed the importance of lattice hydrogen for the synthesis of hydrocarbons [30].

Interface energy calculations

Symmetric (100), (110) and (111) slabs are constructed with the help of the functionalities of pymatgen [24]. In addition, the (5x1) reconstructed (100) and (2x1) reconstructed (110) are considered. Cu(111) and Cu(100) was modelled in (2x2) supercells, and Cu(110) in a (2x1) supercell, such that hydrogen coverages could be scanned by steps of 0.25. K-point grids with a similar in-plane k-point density as for the bulk Cu calculations are used, namely a (10x10x1) grid for (2x2) supercells, (6x16x1) for the (2x1) cells and (4x17x1) for the (5x1) cell. We used slab thicknesses of 14-17 Å (7-8 atomic layers) (see Supplementary Table 2), separated by an equivalent amount of

'vacuum'(dielectric continuum). We tested that Hartree potential and total energies are converged with respect to cell size, which proves that all calculations are in total charge neutral. The accuracy of interface energies is approximately 0.5 meVÅ^{-2}. Furthermore, we apply a dipole correction to the cell [31], such that energies are converged (e.g. if relaxed structures are slightly asymmetric) and add an artificial charge density to the ionic cores with a spread of 0.5 a.u., which is necessary for inhibiting the self-consistent dielectric to enter into core regions where relatively small charge densities can occur. Relaxations were performed partly straight away within the implicit solvation setup, and partly by a prerelaxation in vacuum. As convergence of the electronic scf is harder than for bulk, we increased convergence thresholds partly to 10^{-6} Ry for the electronic scf and 0.001Ry/Å for the forces. After the relaxation of the non-charged system, we performed 10-15 calculations with different number of electrons, where the appropriate range was determined from an analysis of thePZC and an estimated interfacial capacitance of 30 μF cm^{-2}.

We obtain vacuum surface energies that agree up to 2 meVÅ^{-2} with literature values for Cu(100) and Cu(111) (92 and 83 meVÅ^{-2} vs 94 and 82 meVÅ^{-2}[32]). Proton electrosorption was studied for all surfaces but the rather unstable (5x1) reconstructed Cu(100) surface, by adsorbing up to 1 H per surface atom, which we associate with a surface coverage of 1, on all possible adsorption sites (bridge, top, hollow(hcp/fcc site)). Higher coverages than 1 were treated with mixed site adsorption. Three substrate atomic layers and adsorbate atoms were relaxed on both sides of the symmetric slabs, for all calculations. Calculations which did not converge or where hydrogen atoms or molecules desorbed were rejected. CO and mixed CO/H coverages were constructed using different adsorption sites up to a coverage of 0.5 for CO and different H content, up to a full H monolayer. Cu/SCCS-water interface energies are plotted for all calculations in Supplementary Fig. 17 and the results for pristine surfaces tabulated in Supplementary Table 1 and for all finally considered surfaces in Supplementary Table 2.

For the pristine surfaces we find the unreconstructed surfaces mores stable than reconstructed ones, at the potential of zero charge and the expected stability ordering. Furthermore, comparison to the vacuum results indicates that surface energies of pristine surfaces are reduced consistently by approximately 5 meV/atom in SCCS water.

H adsorption

In agreement with the results of others [33], we obtain as most stable H adsorption site the three fold hollow fcc site for Cu(111) and the hollow site for Cu(100). For Cu(100), H adsorption on reconstructed surfaces is favourable up to 75 % coverage, full coverage, however, can only be obtained on unreconstructed surfaces. We found that this is related to strong adsorption on the step sites of the reconstructed surface, weak adsorption, however, in the lower lying (negative step) sites (see

geometries in Supplementary Table 2). It should be noted that the stars in Supplementary Fig. 17 represent the unmodified, as-calculated values for the charge-potential dependence and the interface energy values for pH=0, without $T\Delta S$ correction, as obtained from the Legendre transformed variable charge calculations (described before). It is worth noting that the approach via Legendre transform is equivalent to performing constant Fermi level calculations. We have tested also the agreement with a more direct constant Fermi level approach, where the surface excess charge is iterated in consecutive, standard, constant charge scf calculations towards a target electrode potential (Fermi level). Both methods are equivalent and give equal results. The lines in Supplementary Fig. 17 represent the low order polynomial interpolation with respect to electrode potential Φ, which illustrates the accuracy of the fits. The upper part of Supplementary Fig. 17 illustrates the observed potential-charge dependence together with high order polynomial fits. These fits are only used to determine the interfacial capacitances $d\sigma/d\Phi$. Non-constant interfacial capacitances are due to the self-consistent determination of the dielectric cavity and its dependence on surface charge as already analysed in Ref. [7]. The influence of this problematic behaviour is partly reduced by fitting only a second order polynomial to interfacial energies, as done here.

The great advantage of simulating the electrochemical interface by explicit charging is the possibility of simulating non-trivial pH dependences of interface energies as illustrated in Supplementary Fig. 18, which also includes a comparison to the results as obtained from constant zero net-charge calculations within the Computational Hydrogen Electrode approach [34, 35, 36] (CHE, plotted as dashed grey line) . Surprisingly, in the H adsorption region (regions with negative slopes of the interface energy), no significant differences between the simplified 0-net-charge approach and our approach can be observed . The colored lines fall nearly together with the CHE results. The main difference is the observed curvature of interface energies, especially pronounced for the clean surfaces (potentials larger than the Hydrogen adsorption threshold). This is due to the capacitive charging of the space charge layer and the so-induced dependence of the interface energy of clean metal surfaces on the RHE scale. A potential on the RHE scale is not an absolute potential as e.g. the SHE potential, as it depends on the pH. Indeed different pH values induce different excess surface charges at fixed potential on the RHE scale and thus surface energies that are pH dependent. More details of the methodolgy, the results and interpretation will be published in a separate paper, as they are partly new and not yet commonly applied in the field. A thorough discussion seems too excessive in this Supporting Information file. Other motivation of using approaches beyond the common constant charge calculations can be found in [35] and [36]. The author (N. Hoermann) would be happy to provide more details on request.

CO and CO/H adsorption

As described before we use an extrapolation method to determine the interface energetics for CO covered surfaces, which we do not want to discuss in detail here as we think it necessitates more explanation in form of a separate publication. In general the extrapolation method, though not requiring more computations than the standard CHE (0 net charge calculations) model, allows to include capacitive energy contributions as well as the influence of an adsorbate dependent potential of zero charge, both influences not taken into account in the CHE model. All evaluations that include CO covered surfaces are performed without interpolations between different coverages as this is basically only possible for a single adsorbate when the adsorption energy changes smoothly with coverage. As a result the minimization is performed by comparing the energetics of all considered surfaces. As stated in the text H, CO and CO/H coadsorption all lead to the same conclusions, that Cu interface energies turn negative at approx. -0.4 V vs RHE. In addition to the evaluation in the main text we add below the results for pure CO and CO/H covered surfaces at different CO_2 pressures (CO_2 chemical potentials) in Supplementary Fig. 19. At low pressures (Supplementary Fig. 19 a) H covered and coadsorbed CO/H surfaces play a dominant role, as can be seen by the change in free energy curves for pure CO and CO&H covered surfaces. At high pressures CO adsorption is dominant and CO covered surfaces more stable than mixed and H covered ones, at intermediate pressures mixed coverages dominate. In particular we also added a surface Pourbaix diagram for an intermediate pressure of 1 mbar, to visualize the richness of surface terminations (Supplementary Fig. 20).

As stated in the main text, negative interface formation energies are present, whenever adsorbates lead to an electron exchange with the external circuit. Hence there is no conceptual difference between H(/CO) only and mixed adsorbates. Due to the minimization in eq. 5 adding additional adsorbates and adsorbate configurations, can only lead to smaller (more negative) interface energies. Furthermore the relative independence of general surface energy trends with respect to different CO_2 pressures (Supplementary Fig. 19) ensures that the main conclusions are independent e.g. of the chosen CO chemical potential reference.

Supplementary References

[1] Gao, D. *et al.* Plasma-Activated Copper Nanocube Catalysts for Efficient Carbon Dioxide Electroreduction to Hydrocarbons and Alcohols. *ACS Nano* **11**, 4825–4831 (2017).

[2] Mistry, H. *et al.* Highly selective plasma-activated copper catalysts for carbon dioxide reduction to ethylene. *Nat. Commun.* **7**, 12123– (2016).

[3] Ren, D. *et al.* Selective Electrochemical Reduction of Carbon Dioxide to Ethylene and Ethanol on Copper(I) Oxide Catalysts. *ACS Catal.* **5**, 2814–2821 (2015).

[4] Lum, Y. & Ager, J. W. Stability of Residual Oxides in Oxide-Derived Copper Catalysts for Electrochemical CO_2 Reduction Investigated with ^{18}O Labeling. *Angew. Chem. Int. Ed.* **57**, 551–554 (2018).

[5] Jain, A. *et al.* Commentary: The Materials Project: A materials genome approach to accelerating materials innovation. *APL Mater.* **1**, 011002 (2013).

[6] Gattrell, M., Gupta, N. & Co, A. A review of the aqueous electrochemical reduction of CO_2 to hydrocarbons at copper. *J. Electroanal. Chem.* **594**, 1 – 19 (2006).

[7] Letchworth-Weaver, K. & Arias, T. A. Joint density functional theory of the electrode-electrolyte interface: Application to fixed electrode potentials, interfacial capacitances, and potentials of zero charge. *Phys. Rev. B* **86**, 075140 (2012).

[8] Wang, Y., Zheng, Y., Huang, C. Z. & Xia, Y. Synthesis of Ag Nanocubes 18-32 nm in Edge Length: The Effects of Polyol on Reduction Kinetics, Size Control, and Reproducibility. *J. Am. Chem. Soc.* **135**, 1941–1951 (2013).

[9] Niu, W., Zhang, L. & Xu, G. Shape-Controlled Synthesis of Single-Crystalline Palladium Nanocrystals. *ACS Nano* **4**, 1987–1996 (2010).

[10] Lu, S.-C. *et al.* Single-Crystalline Copper Nano-Octahedra. *Chem. Mater.* **27**, 8185–8188 (2015).

[11] Loiudice, A. *et al.* Tailoring Copper Nanocrystals towards C2 Products in Electrochemical CO_2 Reduction. *Angew. Chem. Int. Ed.* **55**, 5789–5792 (2016).

[12] Andreussi, O., Dabo, I. & Marzari, N. Revised self-consistent continuum solvation in electronic-structure calculations. *J. Chem. Phys.* **136**, 064102 (2012).

[13] Fisicaro, G., Genovese, L., Andreussi, O., Marzari, N. & Goedecker, S. A generalized Poisson and Poisson-Boltzmann solver for electrostatic environments. *J. Chem. Phys.* **144**, 014103 (2016).

[14] ENVIRON package. http://www.quantum-environment.org.

[15] Giannozzi, P. *et al.* QUANTUM ESPRESSO: a modular and open-source software project for quantum simulations of materials. *J. Phys.: Condens. Matter* **21**, 395502 (2009).

[16] Tripkovic, V., Björketun, M. E., Skúlason, E. & Rossmeisl, J. Standard hydrogen electrode and potential of zero charge in density functional calculations. *Phys. Rev. B* **84**, 115452 (2011).

[17] Mathew, K., Sundararaman, R., Letchworth-Weaver, K., Arias, T. A. & Hennig, R. G. Implicit solvation model for density-functional study of nanocrystal surfaces and reaction pathways. *J. Chem. Phys.* **140**, 084106 (2014).

[18] Ringe, S., Oberhofer, H. & Reuter, K. Transferable ionic parameters for first-principles Poisson-Boltzmann solvation calculations: Neutral solutes in aqueous monovalent salt solutions. *J. Chem. Phys.* **146**, 134103 (2017).

[19] Sakong, S., Forster-Tonigold, K. & Groß, A. The structure of water at a Pt(111) electrode and the potential of zero charge studied from first principles. *J. Chem. Phys.* **144**, 194701 (2016).

[20] Dupont, C., Andreussi, O. & Marzari, N. Self-consistent continuum solvation (SCCS): The case of charged systems. *J. Chem. Phys.* **139**, 214110 (2013).

[21] Rogal, J. & Reuter, K. Ab Initio Atomistic Thermodynamics for Surfaces: A Primer. In *Experiment, Modeling and Simulation of Gas- Surface Interactions for Reactive Flows in Hypersonic Flights*, (pp. 2–1 – 2–18). Educational Notes RTO–EN–AVT–142, Paper 2. Neuilly–sur–Seine (2007).

[22] Trasatti, S. The "absolute" electrode potential - the end of the story. *Electrochim. Acta* **35**, 269 – 271 (1990).

[23] Janak, J. F. Proof that $\partial E/\partial n = \epsilon$ in density-functional theory. *Phys. Rev. B* **18**, 7165 (1978).

[24] Ong, S. P. *et al.* Python Materials Genomics (pymatgen): A robust, open-source python library for materials analysis. *Comput. Mater. Sci.* **68**, 314 – 319 (2013).

[25] Lejaeghere, K. *et al.* Reproducibility in density functional theory calculations of solids. *Science* **351** (2016).

[26] Marzari, N., Vanderbilt, D., De Vita, A. & Payne, M. C. Thermal Contraction and Disordering of the Al(110) Surface. *Phys. Rev. Lett.* **82**, 3296–3299 (1999).

[27] Larsen, A. H. *et al.* The atomic simulation environment-a Python library for working with atoms. *J. Phys.: Condens. Matter* **29**, 273002 (2017).

[28] Castelli, I. E., Thygesen, K. S. & Jacobsen, K. W. Calculated Pourbaix Diagrams of Cubic Perovskites for Water Splitting: Stability Against Corrosion. *Top. Catal.* **57**, 265–272 (2014).

[29] Persson, K. A., Waldwick, B., Lazic, P. & Ceder, G. Prediction of solid-aqueous equilibria: Scheme to combine first-principles calculations of solids with experimental aqueous states. *Phys. Rev. B* **85**, 235438 (2012).

[30] Tang, Q. *et al.* Lattice-Hydride Mechanism in Electrocatalytic CO_2 Reduction by Structurally Precise Copper-Hydride Nanoclusters. *J. Am. Chem. Soc.* **139**, 9728–9736 (2017).

[31] Andreussi, O. & Marzari, N. Electrostatics of solvated systems in periodic boundary conditions. *Phys. Rev. B* **90**, 245101 (2014).

[32] Álvarez-Falcón, L., Vines, F., Notario-Estévez, A. & Illas, F. On the hydrogen adsorption and dissociation on Cu surfaces and nanorows. *Surf. Sci.* **646**, 221 – 229 (2016).

[33] Pang, X.-Y., Xue, L.-Q. & Wang, G.-C. Adsorption of Atoms on Cu Surfaces: A Density Functional Theory Study. *Langmuir* **23**, 4910–4917 (2007).

[34] Nørskov, J. K. *et al.* Origin of the Overpotential for Oxygen Reduction at a Fuel-Cell Cathode. *J. Phys. Chem. B* **108**, 17886–17892 (2004).

[35] Hörmann, N. *et al.* Some challenges in the first-principles modeling of structures and processes in electrochemical energy storage and transfer. *J. Power Sources* **275**, 531 – 538 (2015).

[36] Nielsen, M., Björketun, M. E., Hansen, M. H. & Rossmeisl, J. Towards first principles modeling of electrochemical electrode-electrolyte interfaces. *Surf. Sci.* **631**, 2 – 7 (2015).
